# Activation of Cascade Pathway for Oxygen Reduction via 4f‐3d Orbital Ladder‐Driven Dual‐Site Synergy

**DOI:** 10.1002/advs.202514432

**Published:** 2025-10-30

**Authors:** Ruiqi Cheng, Kaiqi Li, Yilin Han, Xiaoqian He, Jin Song, Huanxin Li, Chaopeng Fu

**Affiliations:** ^1^ School of Materials Science and Engineering Shanghai Jiao Tong University Shanghai 200240 P. R. China; ^2^ Electrochemical Innovation Lab Department of Chemical Engineering University College London London WC1E 7JE UK; ^3^ Advanced Propulsion Lab University College London Marshgate London E20 2AE UK; ^4^ Christopher Ingold Laboratory Department of Chemistry University College London 20 Gordon Street London WC1H0AJ UK; ^5^ Department of Applied Biology and Chemical Technology The Hong Kong Polytechnic University Hong Kong 999077 P. R. China

**Keywords:** 4f‐3d orbital ladder, cascade pathway, dual‐site synergistic effect, electrocatalysis, oxygen reduction

## Abstract

The oxygen reduction reaction (ORR) remains a major obstacle in green electrochemical energy conversion, driving the pursuit of cost‐effective noble‐metal‐free catalysts. Transition metal (TM) and rare‐earth (RE) compounds have emerged as promising alternatives. However, their catalytic activity is hindered by sluggish electron transfer and restrictive scaling relationships. Herein, a TM/RE heterostructural catalyst that integrates the complementary features of Fe_3_N's tunable 3d orbitals and spin polarization with CeO_2_’s partially filled 4f orbitals and facile Ce^4+^/Ce^3+^ redox transitions, enabling dual‐phase catalytic participation, is designed. The Fe_3_N/CeO_2_ heterostructure forms a dual‐site catalytic heterointerface, which promotes charge redistribution and optimizes intermediate adsorption. This synergy originates from the 4f‐3d orbital ladder via Ce─O─Fe coordination, enabling directed electron transfer, Fermi level equilibration, and increased carrier density. The interfacial coupling further modulates the Fe spin state, enhances Ce─O covalency, and enriches unpaired electrons, thereby co‐activating both phases and establishing a cascade pathway at the heterointerface that circumvents conventional scaling constraints. The proposed mechanism is further verified by in situ Raman spectroscopy and theoretical calculations. The Fe_3_N/CeO_2_ achieves a half‐wave potential of 0.874 V and delivers a maximum power density of 157.8 mW cm^−2^ in aluminum‐air batteries, outperforming commercial Pt/C and underscoring the application prospects of RE‐based heterostructures for next‐generation energy technologies.

## Graphical abstract

The designed Fe_3_N/CeO_2_ heterostructure constructs a unique 4f‐3d orbital ladder through Ce─O─Fe coordination, enabling dual orbital coupling and activating both phases. The resulting dual‐site synergistic effect at the heterointerface triggers a cascade mechanism that simultaneously breaks conventional scaling relationships in the ORR and facilitates efficient electron transfer, thereby significantly enhancing catalytic performance.

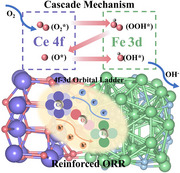

## 1 Introduction

The large‐scale application of renewable and eco‐friendly metal‐air batteries (MABs) remains significantly constrained by challenges associated with the air (oxygen) cathode, where the oxygen reduction reaction (ORR), a complex multi‐electron process, suffers from inherently sluggish kinetics, thereby becoming a major performance‐limiting factor.^[^ ^1^, ^2^ ^]^ Although noble metal electrocatalysts based on platinum‐group elements demonstrate outstanding activity and currently serve as the performance benchmark for ORR, their limited availability and high cost pose serious obstacles to large‐scale implementation.^[^ ^3^, ^4^, ^5^ ^]^ Consequently, to fully realize the commercial potential of these sustainable energy technologies, high‐performance, cost‐effective ORR electrocatalysts must be developed. Rare‐earth (RE) and transition metal (TM) compounds have generated a lot of research attention regarding their multiple redox couplings, versatile electronic configurations, and partially filled 3d/4f electron orbitals.^[^ ^6^, ^7^ ^]^ These properties allow for achieving efficient dissociation of oxygen molecules, yet both categories of materials have similar drawbacks when implemented as electrocatalysts. First, the efficiency of charge transfer during ORR is limited by their semiconducting nature.^[^ ^8^ ^]^ Second, rigid 3d‐2p/4f‐2p interactions between metallic active sites of TM/RE compounds and oxygen impede the adsorption and activation of oxygen species, increasing the overpotentials of ORR.^[^ ^9^ ^]^ Third, large thermodynamic energy barriers resulting from intrinsic scaling relationships among adsorbed oxygen intermediates constrain single‐phase semiconductor‐based electrocatalysts.^[^ ^10^ ^]^

A widely adopted strategy for enhancing the electrocatalytic performance of 4f rare‐earth and 3d transition metal‐based materials involves the deliberate engineering of heterostructures.^[^ ^11^, ^12^ ^]^ These electrocatalysts generally possess heterointerfaces with directed electron flow, regulating charge distribution and the adsorption/desorption of intermediates on metallic active sites, along with increasing carrier concentration for upgraded charge transfer efficiency.^[^ ^13^, ^14^ ^]^ Furthermore, heterointerfaces have the potential to have a dual‐site synergistic effect, lowering the overall energy barrier and overcoming the scaling relations between oxygen intermediates.^[^ ^15^, ^16^ ^]^ Nevertheless, the majority of heterostructure engineering techniques are still empirical, thus rendering it more challenging to figure out the activity origin at the heterointerface. Furthermore, most of these approaches often lack in‐depth discussions of the interfacial bonding modes between phases at the junction, which are vital for comprehending orbital coupling effects and their influence on the effectiveness of electrocatalysis.

Recently, heterostructural electrocatalysts integrating electroactive transition metal (TM) and rare‐earth (RE) compounds have exhibited promising electrocatalytic performance, which is primarily attributed to the internally formed 4f‐3d orbital ladder.^[^ ^17^, ^18^ ^]^ Liu et al initially constructed the 3d‐4f orbital ladders for efficient electrocatalytic water oxidation.^[^ ^17^ ^]^ The Co‐3d‐e_g_ orbital occupancy was optimized through orbital ladder engineering, significantly improving the oxygen evolution performance of Co(OH)_2_. Moreover, Wang et al utilized the construction of TM/RE heterostructure to inhibit the dissolution of Fe atoms and optimize the adsorption behavior of oxygen intermediates, achieving boosted ORR stability and efficiency.^[^ ^19^ ^]^ Subsequently, our group reported that f‐p‐d gradient orbital coupling can effectively enhance the ORR activity, mainly by modulating the spin state of Fe single‐atom active sites.^[^ ^18^ ^]^ Based on the above findings, the following hypothesis can be proposed: through orbital coupling, RE elements, which are distinguished by their strong spin‐orbit coupling, variable 4f valence states, and tunable 4f energy levels close to the Fermi level, can modify TM elements.^[^ ^20^ ^]^ Consequently, the electronic and spin states of 3d transition metal (TM) atoms can be effectively modulated by the 4f‐3d interaction, thereby boosting their activity as catalytic sites. Moreover, RE elements possess 4f^n−1^5d^1^6s^2^ or 4f^n^6s^2^ electronic configurations, enabling transitions between oxidation states (e.g., Eu^3+^/Eu^2+^, Ce^4+^/Ce^3+^). For example, Cerium, the most abundant rare‐earth element (≈0.0046 wt.% of the Earth's crust), readily forms CeO_2_, a high‐temperature O^2−^ ion conductor with exceptional oxygen buffering capacity. This property imparts CeO_2_ with excellent oxygen mobility and regeneration ability, thereby facilitating the adsorption of oxygen intermediates and significantly enhancing its catalytic activity.^[^ ^10^, ^21^, ^22^ ^]^ Additionally, the redistribution of energy levels by the 4f‐3d orbital ladder also facilitates effective electron transport between RE active sites and adsorbed intermediates, lowering overpotential and increasing total activity.^[^ ^17^, ^23^, ^24^ ^]^ This mechanism may also initiate a cascade pathway at heterostructure interfaces, where spatially distinct active sites simultaneously adsorb different oxygen intermediates, thereby breaking conventional scaling relationships and markedly enhancing ORR performance.^[^ ^25^, ^26^ ^]^ Nevertheless, However, most relevant studies have assumed that electrocatalytic reactions occur primarily at well‐defined active sites, overlooking their influence on the adsorption behavior of different intermediates during the reaction. Consequently, the precise role of the 4f‐3d orbital ladder in the ORR still remains insufficiently understood, highlighting the necessity for further investigation into the underlying structure‐activity relationship.

Herein, a Fe_3_N/CeO_2_ heterostructure catalyst was synthesized by electrospinning and subsequent carbonization, where electrostatic interactions between Fe and Ce precursors facilitated heterostructure formation. The catalyst exhibits atomically intimate interfaces that substantially increase carrier concentration and facilitate efficient charge transfer. The Ce─O─Fe coordination at the heterointerfaces induces a 4f‐3d orbital ladder that modulates the electronic structures of both components. This interaction elevates the spin state of Fe in Fe_3_N, increases the charge density on Ce in CeO_2_, and enhances the density of unpaired electrons, thereby activating both phases toward the ORR. This heterostructure, featuring dual active sites, employs a cascade pathway: ORR intermediates preferentially adsorb onto energetically favorable sites. This circumvents conventional scaling relationships and lowers the energy barriers of key ORR steps, enhancing overall efficiency. This culminates in improved ORR performance of the Fe_3_N/CeO_2_ catalyst, exhibiting a half‐wave potential (*E*_1/2_) of 0.874 V that surpasses commercial Pt/C (0.858 V) and demonstrating outstanding promise for MAB applications.

## 2 Results and Discussion

**Figure 1:**
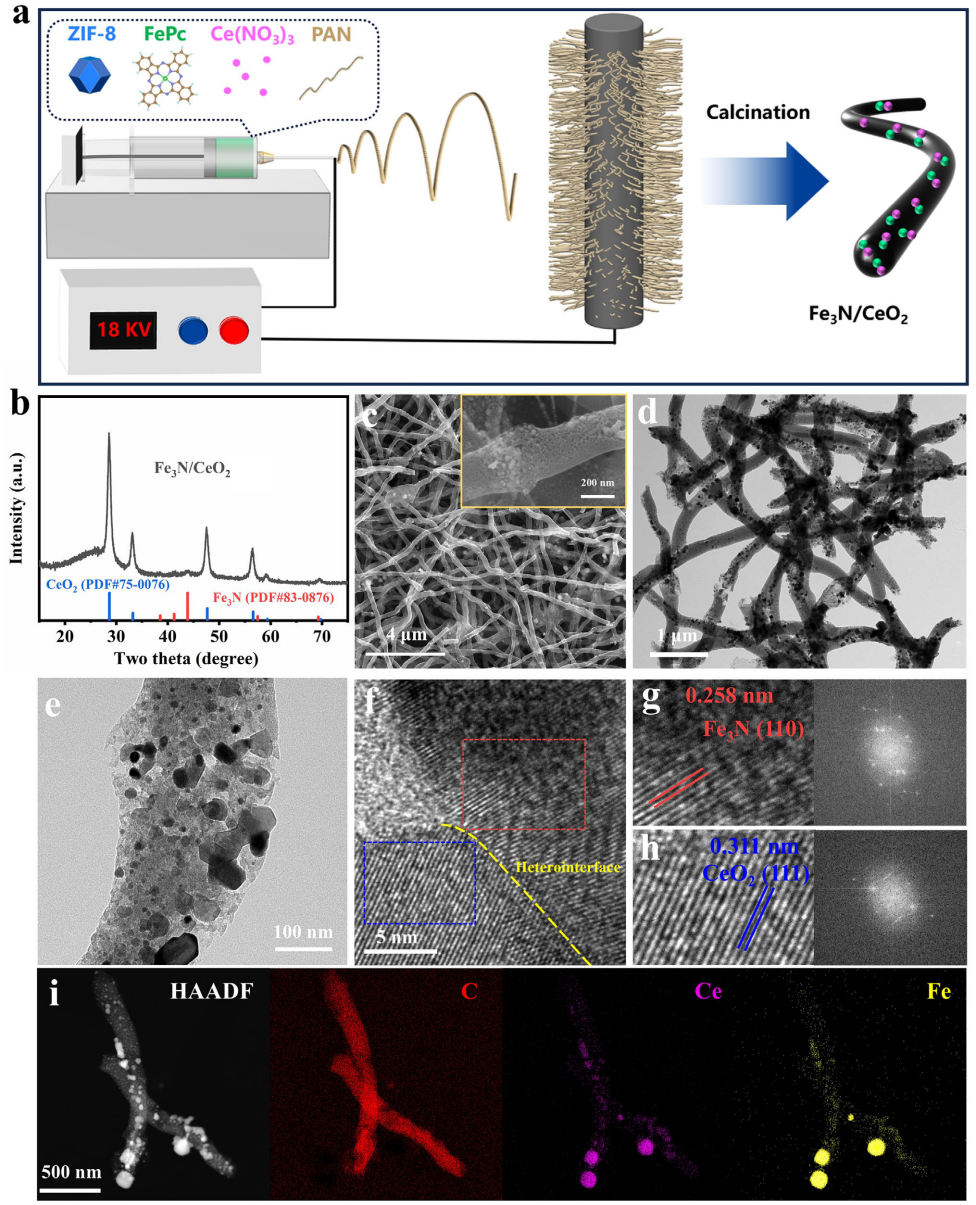
a) Synthetic pathway for Fe_3_N/CeO_2_. b) XRD pattern of Fe_3_N/CeO_2_. c) SEM and d,e) TEM images of Fe_3_N/CeO_2_. f) HRTEM image of heterointerface in Fe_3_N/CeO_2_. Calculation of lattice spacing with corresponding FFT patterns of g) Fe_3_N and h) CeO_2_ phase in Fe_3_N/CeO_2_. i) High‐angle annular dark‐field scanning transmission electron microscopy (HAADF‐STEM) image of Fe_3_N/CeO_2_ with corresponding element mappings of C, Ce, and Fe.

The Fe_3_N/CeO_2_ composite was synthesized via an electrospinning method followed by carbonization (**Figure** **1**a). The π‐electron‐rich phthalocyanine ring creates an electron‐dense environment, promoting electrostatic interactions between FePc and Ce^3+^ in the spinning solution.^[^ ^27^ ^]^ Consequently, the Fe_3_N/CeO_2_ heterostructure was constructed by utilizing the strong coordination ability of FePc and the pronounced Lewis acidity of Ce^3+^. The electron‐rich phthalocyanine ring enhances the affinity between Fe species and the electrophilic Ce^3+^, guiding the formation of the Fe_3_N/CeO_2_ heterostructure during the carbonization process.^[^ ^28^ ^]^ The presence of the Fe_3_N phase (PDF#83‐0876) and the CeO_2_ phase (PDF#75‐0076) in the Fe_3_N/CeO_2_ composite is confirmed by the X‐ray diffraction (XRD) pattern (Figure 1b), indicating the successful formation of both phases from the Fe and Ce precursors. Meanwhile, crystalline structures of Fe_3_N and CeO_2_ can also be confirmed (Figure S1, Supporting Information). Scanning electron microscopy (SEM) images reveal that the as‐spun Fe_3_N/CeO_2_ exhibits an interconnected nanofiber‐like morphology (Figure 1c). The inset in Figure 1c highlights the porous structure of these nanofibers, which arises from the incorporation of ZIF‐8 into the precursor. Notably, control samples prepared without the addition of Ce or Fe sources (denoted as Fe_3_N or CeO_2_, respectively) also display similar nanofibrous architectures with embedded nanoparticles (Figure S2, Supporting Information). Furthermore, energy‐dispersive X‐ray spectroscopy (EDX) elemental maps demonstrate a significant concentration of both Fe and Ce within the Fe_3_N/CeO_2_ composite (Figure S3, Supporting Information). Transmission electron microscopy (TEM) images reveal that nanoparticles are densely and evenly distributed across the porous nanofibers (Figure 1d). Importantly, many nanoparticles appear stacked on the nanofibers, indicating the formation of heterostructures (Figure 1e).^[^ ^29^, ^30^ ^]^ High‐resolution TEM (HRTEM) images of Fe_3_N/CeO_2_ reveal closely contacted heterointerfaces characterized by distinct crystal structures between the constituent phases (Figure 1f). Figure 1g,h, and Figure S4 (Supporting Information) demonstrate lattice spacings of 0.258 and 0.311 nm in the upper and lower regions of Figure 1f, corresponding to the Fe_3_N (110) and CeO_2_ (111) crystal planes, respectively. Meanwhile, the fast Fourier transform (FFT) patterns from these regions reveal differences in crystal structures, further confirming that the boundary between the two phases can be considered a heterointerface in Fe_3_N/CeO_2_. Elemental mappings of Fe_3_N/CeO_2_ clearly show an even distribution of Fe and Ce across the nanoparticles, confirming the successful synthesis and homogeneous dispersion of the Fe_3_N/CeO_2_ heterostructure (Figure 1i). These observations demonstrate the precise assembly of the Fe_3_N/CeO_2_ heterostructure with distinct heterointerfaces at the atomic scale. Additionally, the Fe and Ce contents in the Fe_3_N/CeO_2_ sample were determined by inductively coupled plasma mass spectrometry (ICP‐MS) to be 1.04 wt.% and 1.56 wt.%, respectively. The Ce content in the CeO_2_ sample was measured at 2.38 wt.%, while the Fe content in the Fe_3_N sample was found to be 1.20 wt.%.

The chemical compositions were analyzed by X‐ray photoelectron spectroscopy (XPS). As shown in Figure S5a (Supporting Information), the binding energies of the Fe peaks in Fe_3_N/CeO_2_ are shifted 0.18 eV higher compared to those in Fe_3_N, indicating a decrease in electron density within the iron nitride phase of Fe_3_N/CeO_2_.^[^ ^31^, ^32^ ^]^ Figure S5b (Supporting Information) shows that the Ce 3d peaks in Fe_3_N/CeO_2_ shift to lower binding energies by 0.12 eV compared to those in CeO_2_, indicating an increased charge density in the CeO_2_ phase of Fe_3_N/CeO_2_. By calculating the ratio of Ce^4+^ to Ce^3+^ peaks, the differing oxidation states of cerium in Fe_3_N/CeO_2_ and CeO_2_ were identified. The Ce^4+^/Ce^3+^ ratio for Fe_3_N/CeO_2_ is 3.13, lower than that for CeO_2_ (4.41). These findings indicate that at the Fe_3_N/CeO_2_ heterointerface, the Fe_3_N phase serves as an electron donor and the CeO_2_ phase as an electron acceptor. Upon heterostructure formation, electrons migrate from Fe_3_N to CeO_2_, causing electron accumulation in Ce 2p.

**Figure 2:**
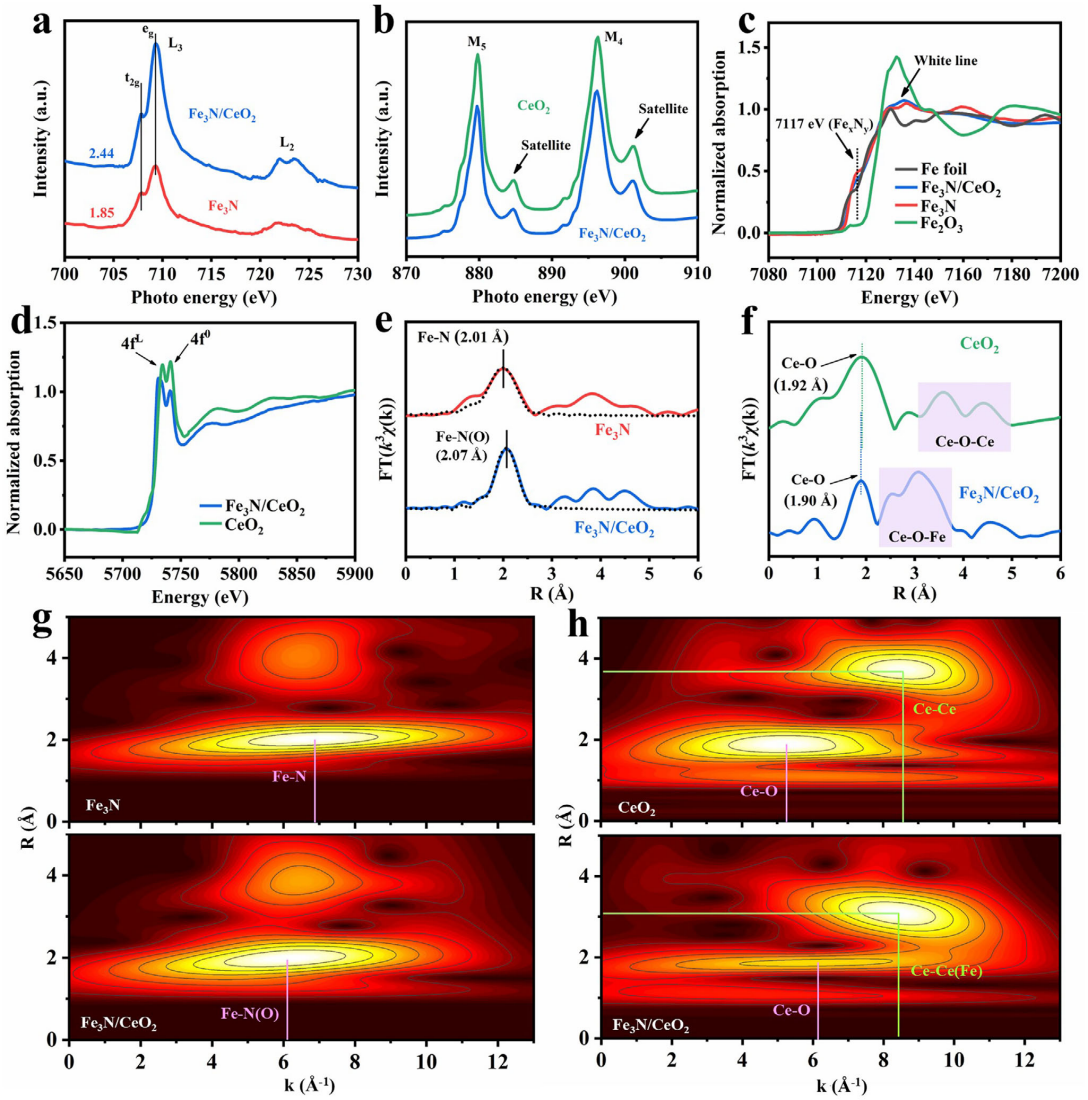
a) Fe L‐edge XANES spectra of Fe_3_N/CeO_2_ and Fe_3_N. b) Ce M‐edge XANES spectra of Fe_3_N/CeO_2_ and CeO_2_. c) Fe K‐edge XANES spectra of Fe_3_N/CeO_2_ and Fe_3_N. d) Ce L_3_‐edge XANES spectra of Fe_3_N/CeO_2_ and CeO_2_. e) Fe K‐edge EXAFS spectra of Fe_3_N/CeO_2_ and Fe_3_N at R space. f) Ce L_3_‐edge EXAFS spectra of Fe_3_N/CeO_2_ and CeO_2_ at R space. g) Fe K‐edge EXAFS WTs contour plots of Fe_3_N/CeO_2_ and Fe_3_N. h) Ce L_3_‐edge EXAFS WTs contour plots of Fe_3_N/CeO_2_ and CeO_2_.

To further elucidate the electronic structure and chemical states, synchrotron‐based X‐ray absorption spectroscopy (XAS) was conducted on Fe_3_N/CeO_2_, Fe_3_N, and CeO_2_ (**Figure** **2**a). The Fe L‐edge X‐ray absorption near‐edge structure (XANES) spectra of both Fe_3_N/CeO_2_ and Fe_3_N display two distinct groups of peaks: the L_3_‐edge peaks (706–712 eV) and the L_2_‐edge peaks (720–726 eV). These correspond to electron transitions from the Fe 2p_3/2_ and Fe 2p_1/2_ levels to the unoccupied Fe 3d orbitals. The L_3_‐edge splitting indicates the energy division of Fe 3d within the crystal field of iron nitride.^[^ ^33^ ^]^ The peaks at 707.7 and 709.2 eV correspond to transitions from the spin‐orbit‐split 2p_3/2_ to the unoccupied t_2g_ low‐energy orbital and e_g_ high‐energy orbital, respectively.^[^ ^34^, ^35^ ^]^ The e_g_ to t_2g_ peak intensity ratio (*I*_eg_/*I*_t2g_) of Fe_3_N/CeO_2_ is 2.44, higher than that of Fe_3_N (1.85), suggesting that heterostructure formation stimulates the influx of more electrons from the t_2g_ orbital (d_xz_ and d_yz_) to the e_g_ orbitals (d_z_^2^), further enhancing the spin state of atomic Fe. Moreover, the intensity ratio of L_3_‐region peaks to L_2_‐region peaks (L_3_/L_2_) can reflect the d‐orbital occupancy of Fe. The L_3_/L_2_ ratio of Fe_3_N/CeO_2_ (3.99) is higher than that of Fe_3_N (3.27), indicating that the formation of the heterostructure decreases the d‐orbital occupancy of Fe in the Fe_3_N phase.^[^ ^36^ ^]^ This reduction favors the optimization of Fe's bonding and antibonding states and enhances the concentration of unpaired electrons.^[^ ^37^ ^]^

The XANES spectra of the Ce M‐edge for Fe_3_N/CeO_2_ and CeO_2_ are shown in Figure 2b. Peaks between 873 and 887 eV correspond to the M_5_‐edge, while those between 891 and 903 eV correspond to the M_4_‐edge.^[^ ^38^ ^]^ Satellite peaks at 884.8 and 901.2 eV in the M_5_ and M_4_ regions are attributed to Ce^4+^ features. The M_5_ to M_4_ peak intensity ratio (M_5_/M_4_) was employed to assess the average oxidation states of Ce in the samples. Generally, this ratio is lower in materials dominated by Ce^4+^ compared to those dominated by Ce^3+^. The M_5_/M_4_ ratio for Fe_3_N/CeO_2_ is 0.807, which is higher than the 0.778 observed for CeO_2_, indicating an increased proportion of Ce^3+^ following heterostructure formation. This transition promotes electron transfer to the vacant 4f orbital of Ce^4+^ (4f^0^), which enhances covalency and reinforces orbital overlap with the O 2p orbitals of adjacent oxygen atoms. As a result, this overlap facilitates better electron mobility between Ce and O, leading to increased electron delocalization.^[^ ^39^ ^]^

The Fe K‐edge XANES spectra of Fe_3_N/CeO_2_ and Fe_3_N, presented in Figure 2c with Fe foil, FeO, and Fe_2_O_3_ as references, display a pre‐edge peak at 7117 eV. This peak is commonly attributed to the 1s → 3d electronic transition in Fe within Fe_x_N_y_, reflecting the splitting of 3d orbitals and the local coordination environment surrounding the Fe atoms.^[^ ^40^ ^]^ The reduced intensity of the pre‐edge peak in Fe_3_N/CeO_2_ compared to Fe_3_N indicates that the formation of the heterostructure induces local crystal field distortions in Fe_3_N, thereby modifying the coordination environment of Fe. Furthermore, the shift toward a more positive pre‐edge feature in Fe_3_N/CeO_2_ suggests an increased oxidation state of Fe, implying strong electronic coupling between the Fe_3_N and CeO_2_ phases. By enabling electron transfer from Fe_3_N to CeO_2_, this interaction improves electron–hole pair separation and increases the concentration of carriers.^[^ ^41^ ^]^ Additionally, the higher white‐line intensity in Fe_3_N/CeO_2_ compared to Fe_3_N indicates the generation of more unpaired electrons and an increase in vacant electronic states, attributed to the reduced d‐orbital occupancy resulting from heterostructure formation.^[^ ^42^ ^]^ The Ce L_3_‐edge XANES spectra of Fe_3_N/CeO_2_ and CeO_2_ are shown in Figure 2d. Compared to CeO_2_, the L_3_‐edge of Fe_3_N/CeO_2_ shifts to lower photon energy, indicating increased electron occupancy in the Ce 4f orbital after heterostructure formation. This confirms that CeO_2_ acts as an electron acceptor, with its Ce 4f orbital accommodating more electrons donated from the Fe_3_N phase. The peak at ≈5734 eV corresponds to the electron transition from Ce 2p to Ce (4f^L^)5d, where L represents an electron transferred from the O 2p to the Ce 4f orbital. The peak at 5741 eV corresponds to the electron transition from Ce 2p to Ce (4f^0^) 5d of Ce^4+^. The intensity of the 4f^L^ peak of Fe_3_N/CeO_2_ is higher than that of the 4f^0^ peak, whereas the reverse is observed for CeO_2_. This suggests that during heterostructure formation, Ce atoms in CeO_2_ receive more electrons from surrounding atoms, thereby increasing the electron transfer rate from Fe_3_N to CeO_2_ and enhancing electron mobility.^[^ ^43^ ^]^ The 4f^L^ and 4f^0^ peak intensities are lower for Fe_3_N/CeO_2_ than for CeO_2_, revealing that the heterostructure formation Fe_3_N/CeO_2_ leads to a shrinkage in the Ce─O bond length in CeO_2_ and induces the overall lattice distortion. This results from the increased covalency, which strengthens the interatomic attraction and enhances the overlap of electron clouds between Ce and O.^[^ ^44^ ^]^

The Fourier‐transformed Fe K‐edge extended X‐ray absorption fine structure (EXAFS) spectrum of Fe_3_N exhibits a prominent peak at 2.01 Å, corresponding to Fe─N coordination (Figure 2e). Notably, Fe_3_N/CeO_2_ exhibits a shifted main peak at 2.07 Å, which is attributed to the formation of Fe─O coordination with a longer scattering path at the heterointerface.^[^ ^45^ ^]^ Electron transfer through this coordination weakens and elongates the original Fe─N bonds due to electron depletion. Furthermore, the fitting results show that the coordination number of Fe in Fe_3_N/CeO_2_ is 5.75, lower than the 5.99 observed in Fe_3_N (Figure S6 and Table S1, Supporting Information), supporting the decreased d‐orbital occupancy of Fe in Fe_3_N/CeO_2_.^[^ ^46^ ^]^ The Ce L_3_‐edge EXAFS spectra shown in Figure 2f indicate that the Ce─O bond length in Fe_3_N/CeO_2_ is 1.90 Å, slightly shorter than that in CeO_2_. This confirms the covalency contraction effect within the CeO_2_ phase, driven by enhanced covalent interactions resulting from the formation of Ce─O─Fe coordination, as evidenced by the doublet near ≈3 Å. Moreover, the markedly higher intensity ratio of the second shell (Ce─O─Ce/Ce─O─Fe) to the first shell (Ce─O) in Fe_3_N/CeO_2_ compared to CeO_2_ highlights the prominent formation of the Fe_3_N/CeO_2_ heterostructure.

The Fe K‐edge EXAFS wavelet transform (WT) contour plots in Figure 2g,h reveal that Fe_3_N/CeO_2_ displays a signal with intensity maxima at ≈6.1 Å^−1^, differing from Fe_3_N (≈6.9 Å^−1^), indicating that heterostructure formation regulates the local electronic behavior of Fe in Fe_3_N/CeO_2_.^[^ ^47^ ^]^ The Ce L_3_‐edge EXAFS WTs contour plots display that the Fe_3_N/CeO_2_ exhibits two strong signals with intensity maxima at ≈6.1 and ≈8.4 Å^−1^, corresponding to Ce─O and Ce─Ce/Fe coordination, respectively. These values differ from those of CeO_2_ (≈5.2 and 8.6 Å^−1^), indicating the mixing interaction of Ce─O─Fe.^[^ ^48^ ^]^ Moreover, the vertical axis positions corresponding to Ce─Fe (3.1 Å) and Ce─Ce (3.7 Å) further highlight the differences in second‐shell coordination between Fe_3_N/CeO_2_ and pristine CeO_2_. Notably, the convergence of intensity maxima for Fe─N and Ce─O signals at ≈6.1 Å^−1^ following heterostructure formation indicates enhanced electronic coupling at the heterointerface.^[^ ^43^ ^]^ These findings demonstrate that the heterostructure induces a distinct coordination environment and electronic configuration for Fe and Ce atoms compared to their single‐phase counterparts, which is expected to favor ORR performance.

**Figure 3:**
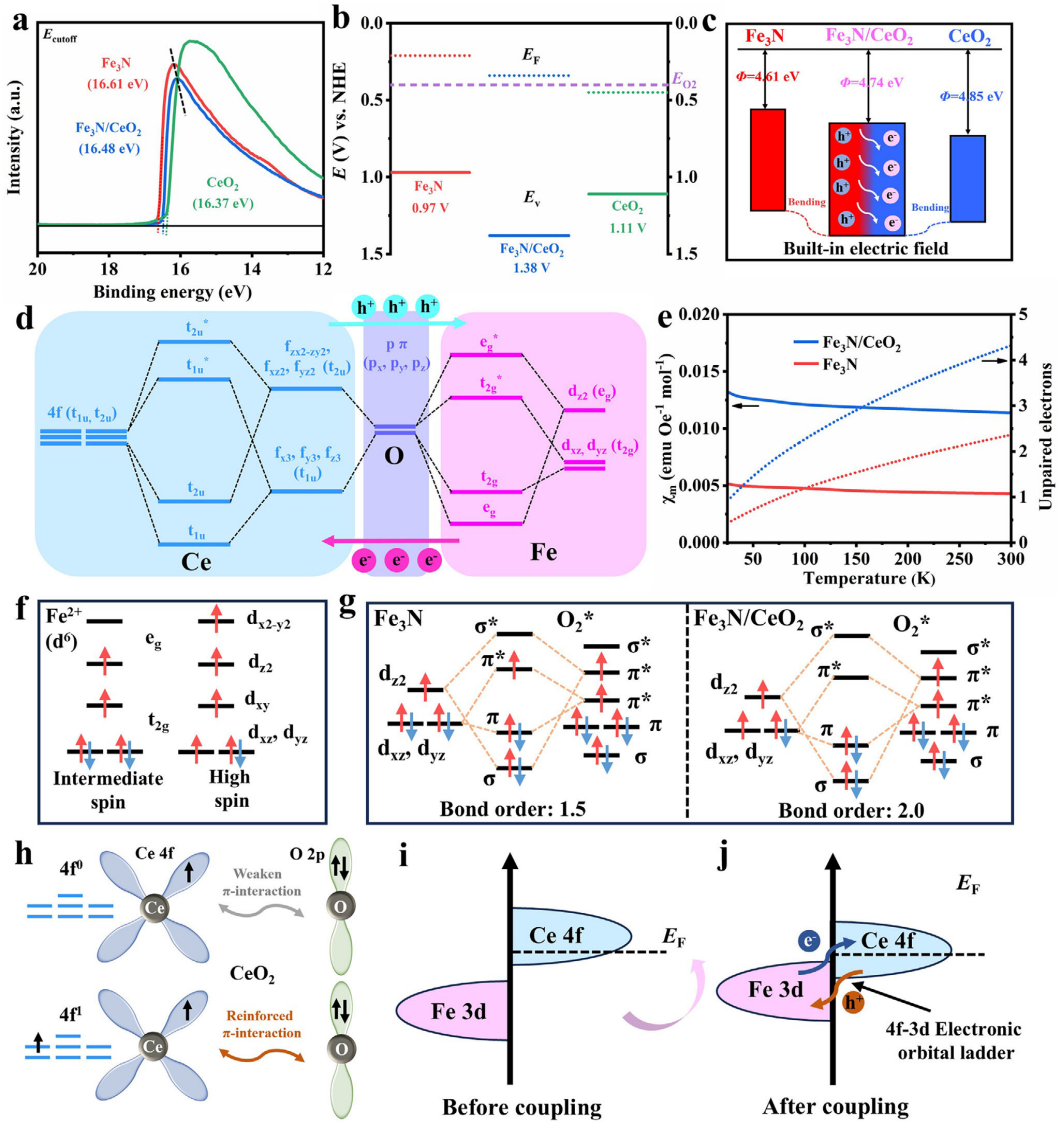
a) UPS spectra (range: 12–20 eV) of Fe_3_N/CeO_2_, Fe_3_N, and CeO_2_. b) Energy band diagrams of Fe_3_N/CeO_2_, Fe_3_N, and CeO_2_. c) Illustration of band bending effect at Fe_3_N/CeO_2_ heterointerface. d) The proposed dual orbital coupling in the Ce─O─Fe coordination. e) ZFC curves of Fe_3_N/CeO_2_ and Fe_3_N. f) d‐electron configurations of Fe^2+^ with intermediate spin state and high spin state. g) Orbital interactions between intermediate spin/high spin Fe^2+^ and O_2_^*^. h) Illustration of interactions between O 2p and Ce 4f^0^/4f^1^ orbitals. i,j) Band center distribution of Fe 3d and Ce 4f before and after coupling.

UV photoelectron spectroscopy (UPS) spectra reveal that the cutoff edges (*E*_cutoff_) of Fe_3_N/CeO_2_, Fe_3_N, and CeO_2_ are 16.48, 16.61, and 16.37 eV, respectively, with corresponding work functions (*Φ*) of 4.74, 4.61, and 4.85 eV (**Figure** **3**a). The *Φ* value of Fe_3_N/CeO_2_, which is higher than that of Fe_3_N but lower than CeO_2_, indicates electron transfer from Fe_3_N to CeO_2_ until Fermi level equilibration is achieved. This results in band bending, with electron accumulation in CeO_2_ and electron depletion (hole‐like behavior) in Fe_3_N. Consequently, numerous electron–hole pairs are generated at the heterointerface. Furthermore, the peak intensity near *E*_cutoff_ for Fe_3_N/CeO_2_ is significantly lower than that for Fe_3_N and CeO_2_, suggesting Fe_3_N/CeO_2_ has the lowest density of states between the Fermi level and the valence band.^[^ ^49^ ^]^ Therefore, the external electric field required for valence electron transition to the Fermi level and subsequently to the O_2_/H_2_O equilibrium potential (*E*_O2_ = 0.401 eV vs NHE) is minimal for Fe_3_N/CeO_2_, leading to the fastest reaction kinetics on this material.^[^ ^50^ ^]^ Moreover, the peak position of Fe_3_N/CeO_2_ is lower than that of Fe_3_N, indicating a shift in the average energy of the d‐orbitals toward the Fermi level.^[^ ^51^ ^]^ This shift can be attributed to the formation of a 4f‐3d orbital ladder, suggesting that Fe_3_N/CeO_2_ likely exhibits a higher (more positive) d‐band compared to Fe_3_N.

Additionally, the measured valence band maxima ​​(*E*_VBM_) of Fe_3_N/CeO_2_, Fe_3_N, and CeO_2_ are 1.04, 0.76, and 0.66 eV, respectively (Figure S7, Supporting Information). The calculated valence band positions (*E*_v_) can be calculated to be 1.38, 0.97, and 1.11 eV, as illustrated in the energy band diagram (Figure 3b). The *E*_v_ of Fe_3_N/CeO_2_ is farther from the O_2_/H_2_O equilibrium potential than those of Fe_3_N and CeO_2_ individually, which can be ascribed to band bending and the formation of an interfacial dipole (Fe_3_N → CeO_2_) via Ce─O─Fe coordination.^[^ ^52^ ^]^ The dual orbital coupling induces band bending and electron redistribution at the heterointerface, decreasing the surface potential of CeO_2_ while increasing that of Fe_3_N, thereby reinforcing the built‐in electric field between them (Figure 3c). This phenomenon not only creates an efficient charge transfer pathway that significantly facilitates electron transport in Fe_3_N/CeO_2_ under the influence of the built‐in electric field but also fosters the spontaneous migration of electrons from the valence band to the unoccupied O 2p (π^*^) orbitals of adsorbed oxygen.^[^ ^53^ ^]^ This effect can regulate the adsorption behavior of oxygen intermediates (e.g., enhancing OOH^*^ adsorption while weakening OH^*^ adsorption), thereby shifting the catalyst closer to the peak of the ORR volcano plot and ultimately improving its thermodynamic catalytic activity.^[^ ^54^ ^]^ This proposed optimization can be attributed to the construction of a 4f‐3d orbital ladder (Figure 3d), which arises from dual orbital coupling in the Ce─O─Fe coordination and enhances electrocatalytic adaptability through more delocalized electronic interactions. After receiving electrons from the Fe_3_N phase, the coupling between 4f orbitals of Ce (t_2u_ (f_x_^3^, f_y_^3^, and f_z_^3^) and t_1u_ (f_zx_^2^_–zy_^2^, f_xz_^2^, and f_yz_^2^)) and 2p orbitals of O (p_x_, p_y_, and p_z_) in the CeO_2_ phase is reinforced due to the increased covalent contribution in Ce─O bonding.^[^ ^55^ ^]^ This effect enhances electron delocalization between Ce and O by contributing to shared electron clouds, facilitating the transport of electrons and holes at the heterointerface, and improving charge transfer efficiency on the CeO_2_ phase.^[^ ^56^ ^]^ Simultaneously, electrons flow out from the Fe_3_N phase, increasing vacant electron states in Fe. This alters its magnetic moment by affecting the unpaired 3d electrons that determine the spin state of Fe.

Subsequently, Zero‐field‐cooled (ZFC) measurements indicate that the number of unpaired electrons at 300 K in Fe_3_N/CeO_2_ reaches 4.32, which is considerably greater than the 2.36 measured in Fe_3_N. This enhancement implies that the spin state of Fe in the Fe_3_N phase shifts from intermediate to high spin after constructing the Fe_3_N/CeO_2_ heterostructure with the 4f‐3d orbital ladder. Based on the measured value of unpaired electrons, the 3d electron configurations of Fe^2+^ in both intermediate and high spin states are demonstrated in Figure 3f. Notably, due to symmetry conservation, the d_xy_ and d_x2–y2_ orbitals of Fe can be disregarded.^[^ ^57^ ^]^ Consequently, only d_xz_, d_yz_, and d_z_^2^ orbitals of FePc are considered for coupling with oxygen intermediates. The calculated bond orders of O_2_^*^ for intermediate spin and high spin Fe are 1.5 and 2.0, respectively (Figure 3g). In particular, the antibonding states, σ^*^ (d_z2_‐p_z_^*^) and π^*^(d_xz_‐p_x_^*^), become less populated following spin‐state enhancement, which strengthens the interaction between Fe and oxygen molecules, thereby reinforcing the subsequent dissociation and further boosting the reaction kinetics.^[^ ^18^ ^]^ Moreover, improved electron transfer on the CeO_2_ phase raises the ratio of Ce^3+^ (4f^1^), significantly strengthening the π‐interaction between Ce and O due to promoted covalency. This optimization improves charge transfer efficiency and increases the binding energy of oxygen intermediates on the CeO_2_ phase (Figure 3h). Within the 4f‐3d orbital ladder at the heterointerface, the f‐band shifts closer to the Fermi level and uplifts the underlying d‐band. This shift enhances the overlap between Fe 3d and Ce 4f orbitals (Figure 3i,j), narrowing the energy gap between the d‐band and f‐band centers and aligning them more closely in energy. This synergy enables complementarity in oxygen intermediate adsorption on dual active sites through a cascade mechanism, balancing the free energy across ORR steps and further reducing the energy barrier.^[^ ^58^ ^]^

**Figure 4:**
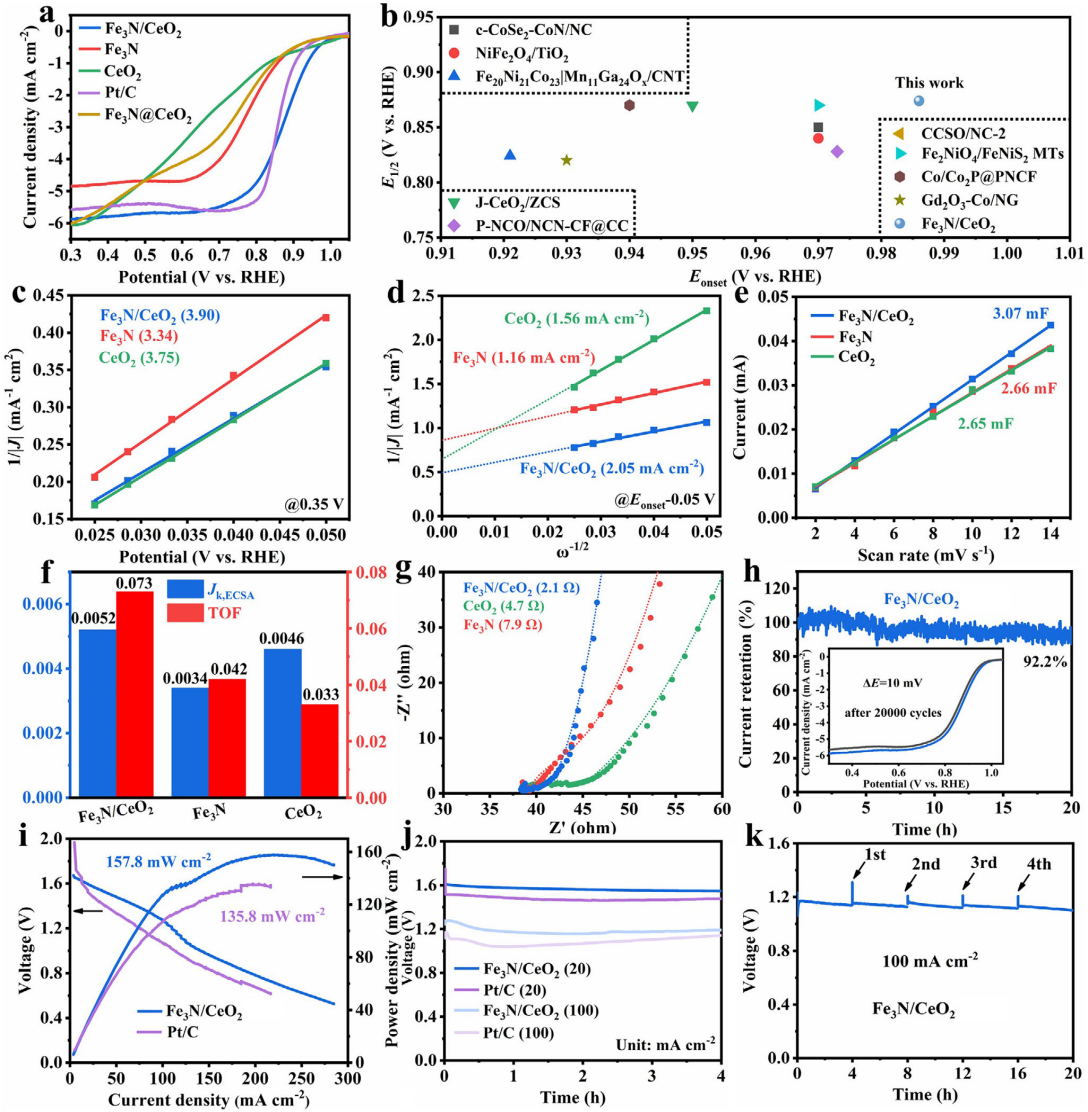
a) LSV curves of Fe_3_N/CeO_2_, Fe_3_N, CeO_2_, Fe_3_N@CeO_2_, and Pt/C. b) *E*_onset_ and *E*_1/2_ comparisons of ORR heterostructural electrocatalysts. Fitted K‐L plots of Fe_3_N/CeO_2_, Fe_3_N, and CeO_2_ for calculating c) *n* and d) *J*_k_. e) *C*_dl_ calculations, f) *J*_k, ECSA_, TOF values, and g) fitted Nyquist plots of Fe_3_N/CeO_2_, Fe_3_N, and CeO_2_. h) Chronoamperometric curves of Fe_3_N/CeO_2_ (inset: LSV curves of Fe_3_N/CeO_2_ before and after 20 000 CV cycles with a scan rate of 0.5 V s^−1^). i) Discharge polarization and power density curves of AABs with Fe_3_N/CeO_2_ and Pt/C. j) Galvanostatic discharge and k) mechanical charging curves of the AABs with Fe_3_N/CeO_2_ and Pt/C.

Electrochemical measurements were performed in an O_2_‐saturated 0.1 m KOH electrolyte to systematically evaluate the electrocatalytic ORR activity of Fe_3_N/CeO_2_. As shown in Figure S8 (Supporting Information), the peak potential (*E*_p_) of Fe_3_N/CeO_2_ (0.901 V) in the cyclic voltammetry (CV) curve is substantially more positive than that of Fe_3_N (0.823 V) and CeO_2_ (0.776 V), suggesting that the ORR is thermodynamically more favorable on Fe_3_N/CeO_2_. The linear sweep voltammetry (LSV) curve in **Figure** **4**a demonstrates that Fe_3_N/CeO_2_ exhibits an onset potential (*E*_onset_) of 0.986 V and a half‐wave potential (*E*_1/2_) of 0.874 V, surpassing those of Fe_3_N (*E*_onset_ = 0.919 V, *E*_1/2_ = 0.783 V) and CeO_2_ (*E*_onset_ = 0.928 V, *E*_1/2_ = 0.640 V) and competing commercial Pt/C (*E*_onset_ = 0.978 V, *E*_1/2_ = 0.858 V), further confirming Fe_3_N/CeO_2_ as the best ORR performer. Moreover, the *E*_onset_ and *E*_1/2_ values of Fe_3_N/CeO_2_ rank among the top in recently reported heterostructural ORR electrocatalysts (Figure 4b; Table S2, Supporting Information). Notably, Fe_3_N/CeO_2_ significantly outperforms mechanically mixed Fe_3_N and CeO_2_ (Fe_3_N@CeO_2_), which possesses *E*_p_, *E*_onset_, and *E*_1/2_ values of 0.819, 0.914, and 0.753 V, respectively. These results indicate that the heterointerface, characterized by the 4f‐3d orbital ladder, plays a critical role in enhancing ORR activity. The fitted Tafel slopes in Figure S9 (Supporting Information) reveal that Fe_3_N/CeO_2_ exhibits the lowest Tafel slope (97.1 mV dec^−1^) compared to other samples, demonstrating its fastest ORR kinetics. Both Fe_3_N/CeO_2_ and commercial Pt/C have Tafel slopes (99.0 mV dec^−1^) within the range of 60–120 mV dec^−1^, indicating that the oxygen reduction process on these two electrocatalysts follows the same dissociation mechanism.^[^ ^59^ ^]^

Furthermore, LSV measurements were performed at rotation speeds ranging from 1600 to 400 rpm (Figure S10, Supporting Information), enabling the calculation of the electron transfer number (*n*) and kinetic current density (*J*_k_) to be determined using the Koutecky–Levich (K‐L) equation. Fitting curves at 0.35 V (diffusion‐controlled region) reveal that the *n* values of Fe_3_N/CeO_2_, Fe_3_N, and CeO_2_ are 3.90, 3.34, and 3.75, respectively (Figure 4c), indicating that the ORR on Fe_3_N/CeO_2_ proceeds via a more efficient four‐electron pathway compared to its counterparts. The *J*_k_ values fitted at *E*_onset_ – 0.05 V (mixed‐control region) are calculated as 2.05, 1.16, and 1.56 mA cm^−2^, respectively (Figure 4d). The largest *J*_k_ of Fe_3_N/CeO_2_ confirms its highest intrinsic ORR activity. Moreover, the fitted double‐layer capacitances (*C*_dl_) of Fe_3_N/CeO_2_, Fe_3_N, and CeO_2_ in non‐Faradaic regions are 3.07, 2.66, and 2.65 mF, respectively (Figure 4e; Figure S11, Supporting Information), with corresponding electrochemically active surface areas (ECSAs) of 76.75, 66.50, and 66.25 cm^2^. Fe_3_N/CeO_2_ exhibits a higher ECSA than Fe_3_N and CeO_2_, which is attributed to the generation of electron–hole pairs at heterointerfaces between Fe_3_N and CeO_2_.^[^ ^50^ ^]^ Moreover, the ECSA‐normalized kinetic current densities (*J*_k, ECSA_) and turnover frequencies (TOF) at 0.79 V were calculated to evaluate the intrinsic activity of a single active site. The *J*_k, ECSA_/TOF of Fe_3_N/CeO_2_, Fe_3_N and CeO_2_ are 0.0052 mA cm^−2^/0.073 s^−1^, 0.0034 mA cm^−2^/0.042 s^−1^, and 0.0046 mA cm^−2^/0.033 s^−1^, respectively (Figure 4f). Fe_3_N/CeO_2_ demonstrates the highest *J*_k, ECSA_, and TOF values among these samples, indicating that the intrinsic activity of active sites in both Fe_3_N and CeO_2_ phases has been significantly improved after forming the Fe_3_N/CeO_2_ heterostructures. This reflects a synergistic effect between the active sites of the two phases at the atomic‐level heterointerface, mediated by the 4f‐3d orbital ladder, which enhances the intrinsic activity of individual active sites. The charge transfer resistances (*R*_ct_) obtained from fitted electrochemical impedance spectroscopy (EIS) plots of Fe_3_N/CeO_2_, Fe_3_N, and CeO_2_ are 2.1, 7.9, and 4.7 Ω, respectively (Figure 4g; Figure S12, and Table S3, Supporting Information). The lowest *R*_ct_ of Fe_3_N/CeO_2_ suggests a greatly enhanced electron transfer rate, attributable to the efficient electron transport pathway at the heterointerface formed by the 4f‐3d orbital ladder. All these factors operate together to provide Fe_3_N/CeO_2_ a superior ORR activity, outperforming that of commercial Pt/C.

The electrochemical stability of Fe_3_N/CeO_2_ in an alkaline electrolyte was also evaluated. The chronoamperometric curve at 0.901 V (CV peak potential) demonstrates a current retention rate of 92.2% after 20 h (Figure 4h). Additionally, the *E*_1/2_ of Fe_3_N/CeO_2_ shows only a 10 mV attenuation after 20 000 continuous CV cycles (inset of Figure 4h). Moreover, the XRD pattern and XPS spectrum of Fe_3_N/CeO_2_ after the chronoamperometric test indicate that the crystal structure remains nearly intact and the chemical state (revealed by the positions of Fe 2p_3/2_ and Fe 2p_1/2_) suffers negligible change following prolonged use (Figures S13 and S14, Supporting Information). TEM images of post‐use Fe_3_N/CeO_2_ further confirm that the heterointerface between Fe_3_N and CeO_2_ still remains observable (Figure S15, Supporting Information). These findings highlight the robust structural stability of Fe_3_N/CeO_2_, demonstrating its exceptional durability in alkaline electrolytes.

Furthermore, alkaline aqueous aluminum‐air batteries (AABs) were assembled using Fe_3_N/CeO_2_ as the cathodic ORR electrocatalyst to evaluate the practical performance of the heterostructural catalyst with a 4f‐3d orbital ladder. As shown in Figure 4i, the AAB with Fe_3_N/CeO_2_ achieves a maximum power density of 157.8 mW cm^−2^, significantly surpassing that of the AAB with commercial Pt/C (135.8 mW cm^−2^) and competitive to those of well‐performed MABs in recent literature (Table S4, Supporting Information). Additionally, the voltage corresponding to the maximum power density is 0.72 V for Fe_3_N/CeO_2_, compared to 0.62 V for Pt/C, confirming the lower internal resistance of Fe_3_N/CeO_2_‐based AAB. This suggests that, owing to the excellent charge transfer efficiency of the Fe_3_N/CeO_2_ heterostructure, AABs with Fe_3_N/CeO_2_ cathodes conduct current more efficiently, thereby reducing energy loss and enabling high power output even at elevated voltages. Moreover, Figure 4j illustrates that the AAB with Fe_3_N/CeO_2_ exhibits a discharge voltage plateau of 1.58 V at a current density of 20 mA cm^−2^, and a plateau of 1.17 V at 100 mA cm^−2^, both higher than those of commercial Pt/C (1.51 and 1.12 V, respectively). To evaluate stability, the AAB with Fe_3_N/CeO_2_ was discharged at 100 mA cm^−2^ for 20 h, with the aluminum anode and electrolyte replaced every 4 h over 4 cycles. The test results show a voltage decay of only 0.06 V, yielding a voltage retention rate of 94.9% (Figure 4k). These findings demonstrate that Fe_3_N/CeO_2_ surpasses commercial Pt/C in both discharge performance and long‐term stability, confirming its practicality and reliability in sustainable energy devices.

**Figure 5:**
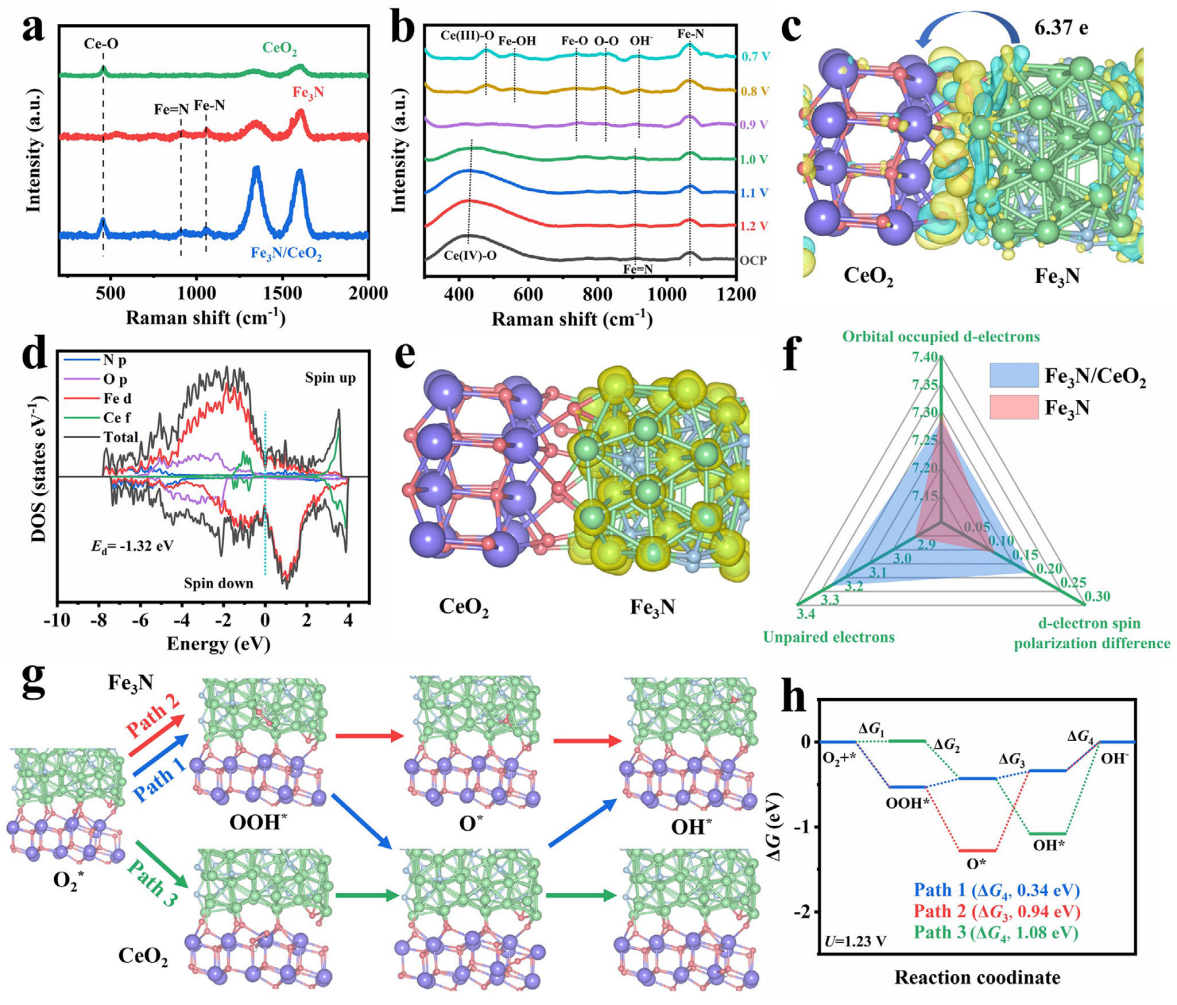
a) Ex situ Raman spectra of Fe_3_N/CeO_2_, Fe_3_N, and CeO_2_. b) In situ Raman spectra of Fe_3_N/CeO_2_ ranging from 1.2 to 0.7 V (vs RHE). c) Charge distribution (yellow: electron increase, cyan: electron decrease), d) density of states, and e) electron spin density (yellow: positive spin, cyan: negative spin) of Fe_3_N/CeO_2_. f) Radar chart of d‐orbital electronic configuration parameters of Fe_3_N/CeO_2_ and Fe_3_N. g) Set ORR paths with optimized configurations of Fe_3_N/CeO_2_ after adsorbing OOH^*^, O^*^, and OH^*^ on the Fe_3_N or CeO_2_ phase. h) Free energy diagram at *U*=1.23 V of different ORR paths.

The ex situ Raman spectra in **Figure** **5**a reveal peaks at 910 and 1060 cm^−1^ observed for Fe_3_N/CeO_2_ and Fe_3_N, corresponding to the stretching of Fe═N and Fe─N bonds, respectively.^[^ ^60^, ^61^ ^]^ Additionally, the peak at 450 cm^−1^ for Fe_3_N/CeO_2_ and CeO_2_ signifies the bending mode of Ce(IV)‐O.^[^ ^62^ ^]^ The ORR on Fe_3_N/CeO_2_ was further analyzed by in situ Raman test at potentials from 1.2 to 0.7 V in 0.1 V intervals (Figure 5b). In the absence of an applied potential, no ORR occurred, and characteristic peaks corresponding to Ce(IV)‐O, Fe═N, and Fe─N were observed. As the potential decreased from 1.2 to 1.0 V, the Ce(IV)‐O peak redshifted with reduced intensity, attributed to charge redistribution at heterointerfaces caused by electron transfer from Fe_3_N to CeO_2_ during circuit connection.^[^ ^63^ ^]^ This redistribution enhances charge transfer efficiency within the Fe_3_N/CeO_2_ heterostructure during subsequent ORR processes.^[^ ^64^ ^]^ At 0.9 V, the ORR was activated, resulting in a significant drop in the Ce(IV)‐O peak intensity, signaling the initiation of oxygen adsorption on CeO_2_ (Equation 1). Meanwhile, the Fe═N peak diminished while the Fe─N peak persisted, suggesting Fe═N acted as the active ORR site. The disappearance of Fe═N was accompanied by two new peaks at 740 cm^−1^ (Fe─O in Fe─OOH^*^) and 825 cm^−1^ (O─O in Fe─OOH^*^),^[^ ^65^, ^66^, ^67^ ^]^ indicating Fe_3_N captured O_2_^*^ from CeO_2_ and converted it to OOH^*^ (Equation 2), accelerating the protonation of oxygen. At 0.8 V, two peaks at 480 and 560 cm^−1^ emerged, corresponding to Ce(III)‐O and Fe─OH, respectively.^[^ ^62^, ^68^, ^69^ ^]^ This suggests that OOH^*^ is readily converted to O^*^ on CeO_2_ (Equation 3), while O^*^ is converted to OH* on Fe_3_N (Equation 4). A peak at 920 cm^−1^ representing OH^−^ confirms the facile desorption of OH^*^ (OH^*^→OH^−^, Equation 5).^[^ ^62^ ^]^ At 0.7 V, the intensities of all oxygen species peaks increased, indicating the accumulation of oxygen intermediates on the catalyst surface as the potential shifted negatively.^[^ ^53^, ^70^ ^]^

(1) $$\mathrm{Ce}\;+O_{2\;}+*\;\to \mathrm{Ce}-O_{2}*$$

(2)

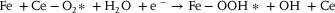

(3) $$\mathrm{Ce}\;+\mathrm{Fe}-\mathrm{OOH}*\;+e^{-}\to \mathrm{Ce}-O*\;+OH^{-}+\mathrm{Fe}$$

(4)

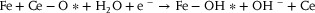



(5)

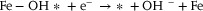




The analysis confirms that, in Fe_3_N/CeO_2_, oxygen intermediates preferentially adsorb at the most favorable active sites across the two phases for subsequent reactions. Specifically, O_2_ and OOH^*^ preferentially react on CeO_2_, whereas O_2_^*^ and O^*^ preferentially react on Fe_3_N. Throughout the ORR process, oxygen species can repeatedly alternate between phases, thereby minimizing the energy barrier at each intermediate step and indicating a cascade mechanism.^[^ ^71^ ^]^ Such synergistic effects between multiple active sites in the heterostructure can effectively break the scaling relationship between the adsorption energies of different intermediates, thus achieving higher ORR activity.

To further substantiate the observed results above from a theoretical perspective, density functional theory (DFT) calculations were performed. In the heterostructural configuration of Fe_3_N/CeO_2_ (Figure 5c), the Fe_3_N phase functions as an electron donor, transferring 6.37 e Bader charge to the CeO_2_ phase. This charge transfer leads to electron accumulation on the CeO_2_ side and hole accumulation on the Fe_3_N side of the heterointerface. The presence of ejected electrons in the CeO_2_ phase contributes to the strengthened Ce─O covalency.^[^ ^43^ ^]^ Moreover, significant charge redistribution is observed at the heterointerface, indicating strong electronic coupling within the 4f‐3d ladder. The density of states (DOS) analysis reveals an upward shift in the d‐band center (*E*_d_) of the Fe_3_N phase from −1.35 to −1.32 eV after forming the 4f‐3d ladder (Figure 5d; Figure S16, Supporting Information), which facilitated the adsorption of oxygen and reduced activation energy, correlating with the analysis in Figure 3g.^[^ ^72^ ^]^ Furthermore, this shift brings the d‐band closer to the Fermi level and reduces the d‐orbital occupancy, stimulating the generation of unpaired electrons and enhancing electron mobility.^[^ ^73^ ^]^

The electron spin density shown in Figure 5e indicates that spin exists exclusively on Fe atoms and is entirely enriched in the positive spin, confirming their high spin state. To explore the impact of the 4f‐3d ladder formation on electron and spin states, the partial density of states (PDOS) of Fe was analyzed (Figure S17, Supporting Information). The orbital‐occupied d‐electrons and unpaired electrons of Fe_3_N/CeO_2_ were calculated to be 7.28 and 3.26, respectively, which are lower and higher than those of Fe_3_N (7.30 and 2.91), respectively (detailed calculations provided in the Supporting Information). This result supports the more balanced bonding/antibonding state and increased carrier concentration in Fe_3_N/CeO_2_.^[^ ^18^ ^]^ Notably, the difference between spin‐up and spin‐down d‐electrons in Fe_3_N/CeO_2_ is calculated as 0.182, exceeding that of Fe_3_N (0.111), which confirms an enhanced spin state of Fe species in Fe_3_N/CeO_2_. This effect originates from the f‐band splitting of Ce, also attributed to the formation of the 4f‐3d ladder. As shown in Figure S18 (Supporting Information), the Ce f‐band at ≈1.15 eV splits into two bands at ≈−1.0 and 3.5 eV after forming the heterostructure. The band at ≈−1.0 eV overlaps with the Fe d‐band, optimizing its electron and spin states and enabling easier electron migration between Fe and Ce at the heterointerface. In this context, electrons may freely transfer between oxygen intermediates adsorbed on Fe_3_N and CeO_2_ during ORR, further validating the speculated cascade mechanism observed in the in situ Raman spectra.

The ORR pathway at the Fe_3_N/CeO_2_ heterointerface was simulated to confirm the proposed cascade mechanism. Initially, oxygen is adsorbed onto CeO_2_ for subsequent reduction. The expected pathway aligns with the Raman results (Path 1), while two additional pathways were simulated with oxygen intermediates (OOH^*^, O^*^, and OH^*^) adsorbed exclusively on either the Fe_3_N or CeO_2_ phase (Path 2 or Path 3). Notably, the free energy changes across the four steps (Δ*G*_1_‐Δ*G*_4_) in Path 1 are significantly more balanced (Figure S19, Supporting Information). This is attributed to the role of the 4f‐3d ladder, which facilitates O^*^ hydroxylation on CeO_2_ during step 3 (O^*^ → OH^*^) and avoids peroxide formation while enabling easier desorption of OH^*^ on Fe_3_N during step 4 (OH^*^ → OH^−^). Together, these processes ensure a cascade pathway originating from the dual‐site synergistic effect. The calculated energy barrier for ORR via Path 1 is 0.34 eV, substantially lower than those for Path 2 (0.94 eV) and Path 3 (1.08 eV). These findings validate that the synergistic dual‐site synergy with a cascade mechanism, driven by the 4f‐3d orbital ladder, effectively breaks the scaling relationship and achieves superior activity toward ORR (Figure 5h).

## 3 Conclusion

In summary, the Fe_3_N/CeO_2_ heterostructural catalyst synthesized via electrospinning exhibits outstanding ORR performance in alkaline media, achieving a half‐wave potential of 0.874 V and a peak power density of 157.8 mW cm^−2^ in alkaline AABs, surpassing the benchmark Pt/C catalyst. This enhanced activity originates from the formation of a 4f‐3d orbital ladder at the Fe_3_N/CeO_2_ heterointerface, where interfacial electron transfer from Fe_3_N to CeO_2_ induces charge redistribution and band structure optimization. The consequent increase in the Fe spin state and the strengthened Ce─O covalency activate both components, enhancing charge transport and enabling a dual‐site synergistic effect via Ce─O─Fe coordination that initiates a cascade pathway toward the ORR. In situ Raman spectroscopy provides direct experimental evidence of the cascade mechanism, wherein reaction intermediates preferentially adsorb at energetically favorable sites due to the increased diversity of highly active centers. Furthermore, density functional theory calculations confirm that this cascade pathway breaks traditional scaling relationships and lowers the overall energy barrier to 0.34 eV, thereby enhancing catalytic efficiency. These findings not only establish Fe_3_N/CeO_2_ as a high‐performance ORR catalyst but also provide fundamental guidance for the rational design of cost‐effective heterostructural electrocatalysts through dual orbital coupling between 3d transition metal and 4f rare‐earth elements.

## Conflict of Interest

The authors declare no competing financial interest.

## Supporting information



Supporting Information

## Data Availability

The data that support the findings of this study are available from the corresponding author upon reasonable request.
