# Supplementary material for: Activation of Cascade Pathway for Oxygen Reduction via 4f‐3d Orbital Ladder‐Driven Dual‐Site Synergy
Source: Adv Sci (Weinh). 2025 Oct 30;13(4):e14432. doi: 10.1002/advs.202514432 (PMC12822467; doi:10.1002/advs.202514432)
Supplement: Supplementary file 1 — Supporting Information [file ADVS-13-e14432-s001.docx]

**Supporting Information**

**Activation of Cascade Pathway for Oxygen Reduction via 4f-3d Orbital Ladder-Driven Dual-Site Synergy**

*Ruiqi Cheng, Kaiqi Li, Yilin Han, Xiaoqian He, Jin Song, Huanxin Li^*^, and Chaopeng Fu^*^*

Dr R. Cheng, Y. Han, X. He, J. Song, Prof. C. Fu

School of Materials Science and Engineering

Shanghai Jiao Tong University

Shanghai 200240, P. R. China

E-mail: [chaopengfu@sjtu.edu.cn](mailto:chaopengfu@sjtu.edu.cn) (Prof. Chaopeng Fu)

Dr H. Li

Electrochemical Innovation Lab

Department of Chemical Engineering

University College London

London WC1E 7JE, United Kingdom

E-mail: [huanxin.li@ucl.ac.uk](mailto:huanxin.li@ucl.ac.uk) (Dr. Huanxin Li)

Dr H. Li

Advanced Propulsion Lab

University College London

Marshgate, London, E20 2AE, United Kingdom

K. Li

Christopher Ingold Laboratory

Department of Chemistry

University College London

20 Gordon Street, London WC1H0AJ, United Kingdom

Dr R. Cheng

Department of Applied Biology and Chemical Technology

The Hong Kong Polytechnic University

Hong Kong, 999077, P. R. China

**Methods**

**Synthesis of ZIF-8**

0.558 g of Zn(NO_3_)_2_·6H_2_O and 0.616 g of 2-methylimidazole were dissolved in 15 mL of methanol to make solutions A and B, respectively. After stirring the mixture of solutions A and B for 24 h, the product was collected by centrifugation, washed with ethanol, and then dried in a vacuum oven for 24 h to obtain ZIF-8.

**Synthesis of Fe_3_N/CeO_2_**

0.176 g of Ce(NO_3_)_3_·6H_2_O and 0.2 g of FePc were dissolved in 5 mL of DMF, and then 0.1 g of ZIF-8 was added and stirred for 4 h to form a uniform mixture. Next, 0.6 g of polyacrylonitrile (PAN, molecular weight: 150000) was slowly added and stirred for 24 h to form a gel-like suspension. The suspension was then transferred to a 5 mL syringe with a 20-gauge stainless steel needle for electrospinning, where the syringe was pushed at a rate of 0.4 mL h^-1^, and the distance between the needle and the rotating collector with aluminum foil (200 rpm) was 20 cm and the operating voltage was 18 kV. The obtained polymer nanofibers were first heated at 250°C for 2 h in the air in a muffle furnace for stabilization, and then calcinated at 800°C at a rate of 5°C min^-1^ in N_2_ in a tube furnace for 3 h to obtain the final product Fe_3_N/CeO_2_. In addition, Fe_3_N and CeO_2_ catalysts were prepared using the same procedures but without adding Ce(NO_3_)_3_·6H_2_O or FePc in the first step.

**Material characterizations**

X-ray diffraction (XRD) was conducted on an Ultima IV X diffractometer. Scanning electron microscopy (SEM) and transmission electron microscopy (TEM) images were obtained on an Apero 2C SEM and a TALOS F200X TEM, respectively. X-ray photoelectron spectroscopy (XPS) and ultraviolet photoelectron spectroscopy (UPS) were performed on an AXIS UltraDLD spectrometer. Raman spectra were collected on a Renishaw inVia Qontor confocal Raman microscope. The temperature-dependent magnetic susceptibility in zero-field-cooled (ZFC) curves were collected under the magnetic field of 2000 Oe from 2 K to 300 K (the temperature gradient was set as 0.1 K). The X-ray absorption spectra (XAS) of Fe K-edge and Ce L-edge were collected using hard X-ray at the BL17B1 beamline of Shanghai Synchrotron Radiation Facility (SSRF) and the XAS of Fe L-edge and Ce M-edge were collected using soft X-ray at the BL02B02 beamline of SSRF.

**Electrochemical tests**

All electrochemical tests were carried out in a three-electrode system in an O_2_-saturated 0.1 M KOH electrolyte, where the reference electrode was a saturated calomel electrode (SCE), the counter electrode was a graphite rod, and the working electrode was a catalyst-modified glassy carbon rotating disk electrode (GC RDE). All potentials were calibrated to the reversible hydrogen electrode (RHE) according to the Nernst equation (*E*_RHE_ = *E*_SCE_+ 0.2412+ 0.0592*pH, unit: V). The CV and LSV curves were measured at a scan rate of 10 mV s^-1^. During the cyclic test, the potential window was set from 1.2 V to 0.6 V, and the scan rate was set at 50 mV s^-1^.

The catalyst ink was obtained by dispersing 4 mg of the catalyst in a mixture of 360 μL ethanol, 120 μL deionized water, and 20 μL 5% Nafion solution and ultrasonicating for 1 h, and then 14 μL of the catalyst ink was deposited on the GC RDE (area = 0.19625 cm^2^). The catalyst-modified GC RDE was fixed on a rotating shaft to form a uniform, thin, and smooth catalyst layer during air drying.

**Aqueous aluminum-air battery (AAB) tests**

The Fe_3_N/CeO_2_ catalyst, XC-72 carbon black, and polytetrafluoroethylene (PTFE, 60% dispersion) were dispersed in ethanol at a ratio of 1:3:1. After removing excess solvent, the obtained paste was repeatedly rolled and coated on a nickel foam current collector to prepare the air cathode. The anode and the electrolyte were Al-1.0 wt% Mg-0.1 wt% Sn (Al-Mg-Sn) alloy and 4 M NaOH, respectively. The anode and electrolyte of AAB were refreshed for mechanical charging every 4 hours. All battery tests were performed on a LAND testing system (LAND Electronics Ltd.).

**Density functional theory (DFT) calculations**

The first-principles tool——Vienna Ab initio Simulation Package(VASP) was employed to perform all density functional theory (DFT) calculations within the generalized gradient approximation (GGA) using the Perdew-Burke-Ernzerhof (PBE) formulation.^[1]^ The projected augmented wave (PAW) potentials were adopted to describe the ionic cores and take valence electrons into account using a plane wave basis set with a kinetic energy cutoff of 450 eV.^[2]^ Partial occupancies of the Kohn−Sham orbitals were allowed using the Gaussian smearing method and a width of 0.05 eV. For the optimization of both geometry and lattice size, the Brillouin zone integration was performed with 2×2×1 *Γ*-centered *k*-point sampling.^[3]^ The self-consistent calculations applied a convergence energy threshold of 10^-5^ eV. The equilibrium geometries and lattice constants were optimized with maximum stress on each atom within 0.04 eV Å^-1^. The 15 Å vacuum layer was normally added to the surface to eliminate the artificial interactions between periodic images. The weak interaction was described by the DFT+D3 method using empirical correction in Grimme’s scheme.^[4]^ The spin polarization method was adopted to describe the magnetic system. Moreover, the input files and output data of charge difference were generated by the tool——Vaspkit.^[5]^

To consider the strong correlation effects of transition metal in structure, both structural optimizations and electronic structure calculations were carried out by using the spin-dependent GGA plus Hubbard correction *U* method, and the effective *U*_eff_ parameter is 5.0 eV for Ce atoms. The free energy (Δ*G*) of each step was calculated according to the following equation:

Δ*G* = Δ*E*_ZPE_ + Δ*E* – *T*Δ*S*

Where Δ*E*_ZPE_ is the zero-point energy at 298.15 K; Δ*E* is the binding energy of the intermediates; T is the experimental temperature (298.15 K); and Δ*S* is the entropy change. The Bader charge analysis was also applied to this work.^[6]^ The first oxygen adsorption step (O_2_ + * → O_2_*) was set on the CeO_2_ phase, and the reaction coordinate can be simulated based on the following equation:

The in-situ Raman spectra were collected in a home-made electrochemical cell with reference electrode and counter electrode reported in our previous work.^[7]^ During the spectra collection, oxygen was slowly bubbled into the electrolyte to ensure saturation of oxygen in the electrolyte. The applied potential shifts from 1.2 V to 0.7 V in 0.1 V intervals. Each spectrum was collected after switching the external potential for 600 s. The alternative ORR pathway based on in-situ Raman spectra (Path 1) is as follows: #represents the Ce sites on CeO_2_, *represents the Fe sites on Fe_3_N.

O_2_# + H_2_O + e^-^ → OOH* + OH^-^

OOH* + e^-^ → O# + OH^-^

O# + H_2_O + e^-^ → OH* + OH^-^

OH* + e^-^ → * + OH^-^

The ORR pathway solely on the Fe_3_N or CeO_2_ side site (Path 2 and Path 3) is as follows: *represents the Fe sites on Fe_3_N or Ce sites on CeO_2_.

O_2_* + H_2_O + e^-^ → OOH* + OH^-^

OOH* + e^-^ → O* + OH^-^

O* + H_2_O + e^-^ → OH* + OH^-^

OH* + e^-^ → * + OH^-^





**Figure S1.** (b) XRD patterns of Fe_3_N and CeO_2_.


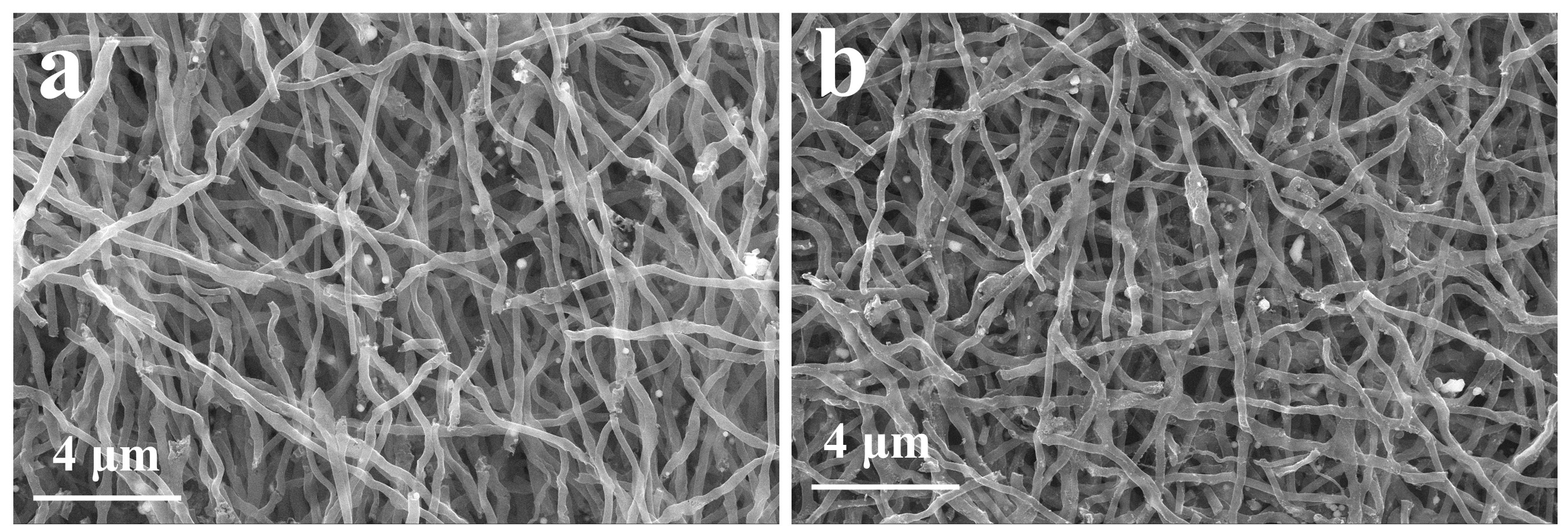


**Figure S2.** SEM images of (a) Fe_3_N and (b) CeO_2_.


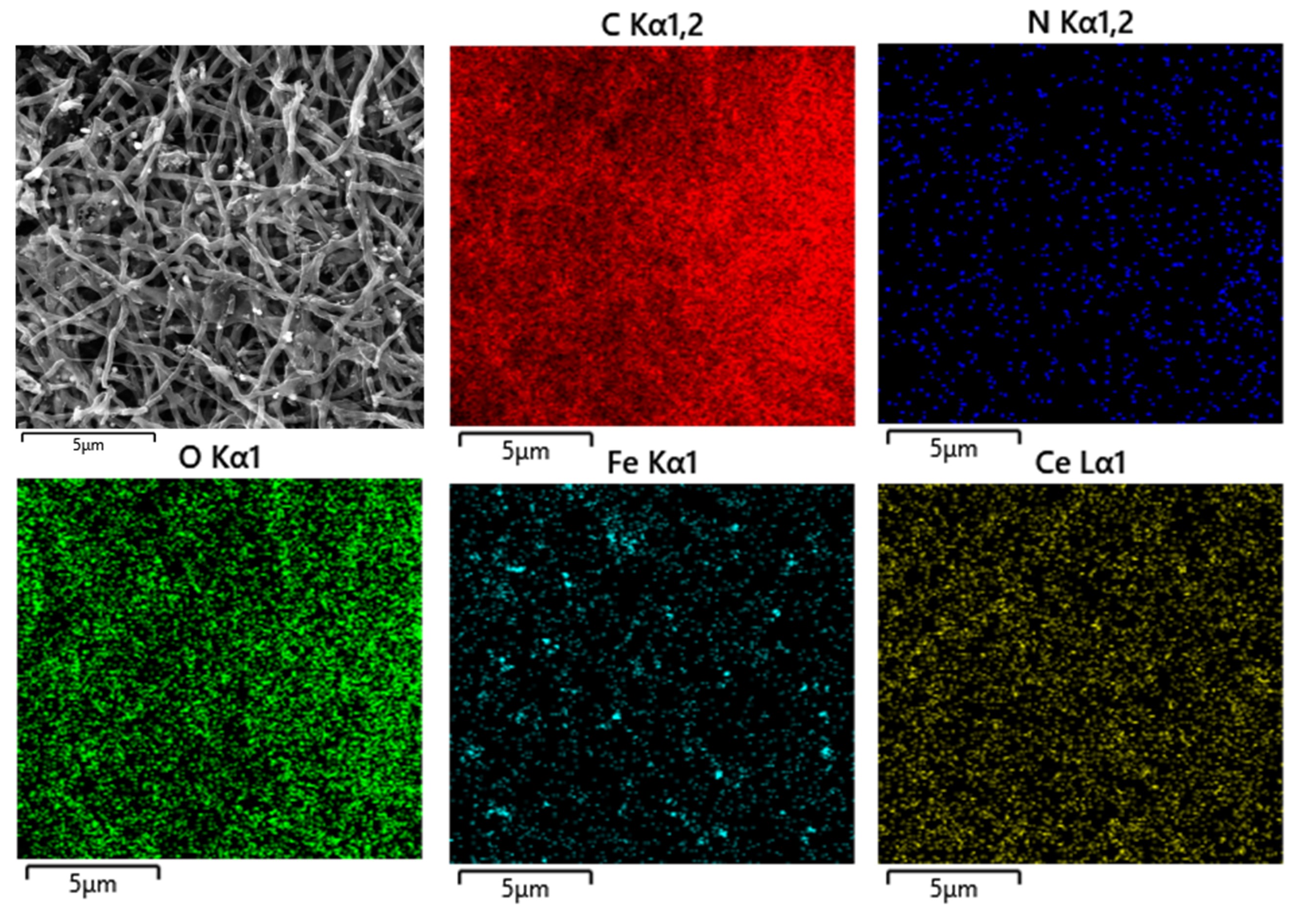


**Figure S3.** SEM image with EDX element mappings of Fe_3_N/CeO_2_.





**Figure S4.** Lattice spacings calculated from the upper and lower regions of the heterointerface.


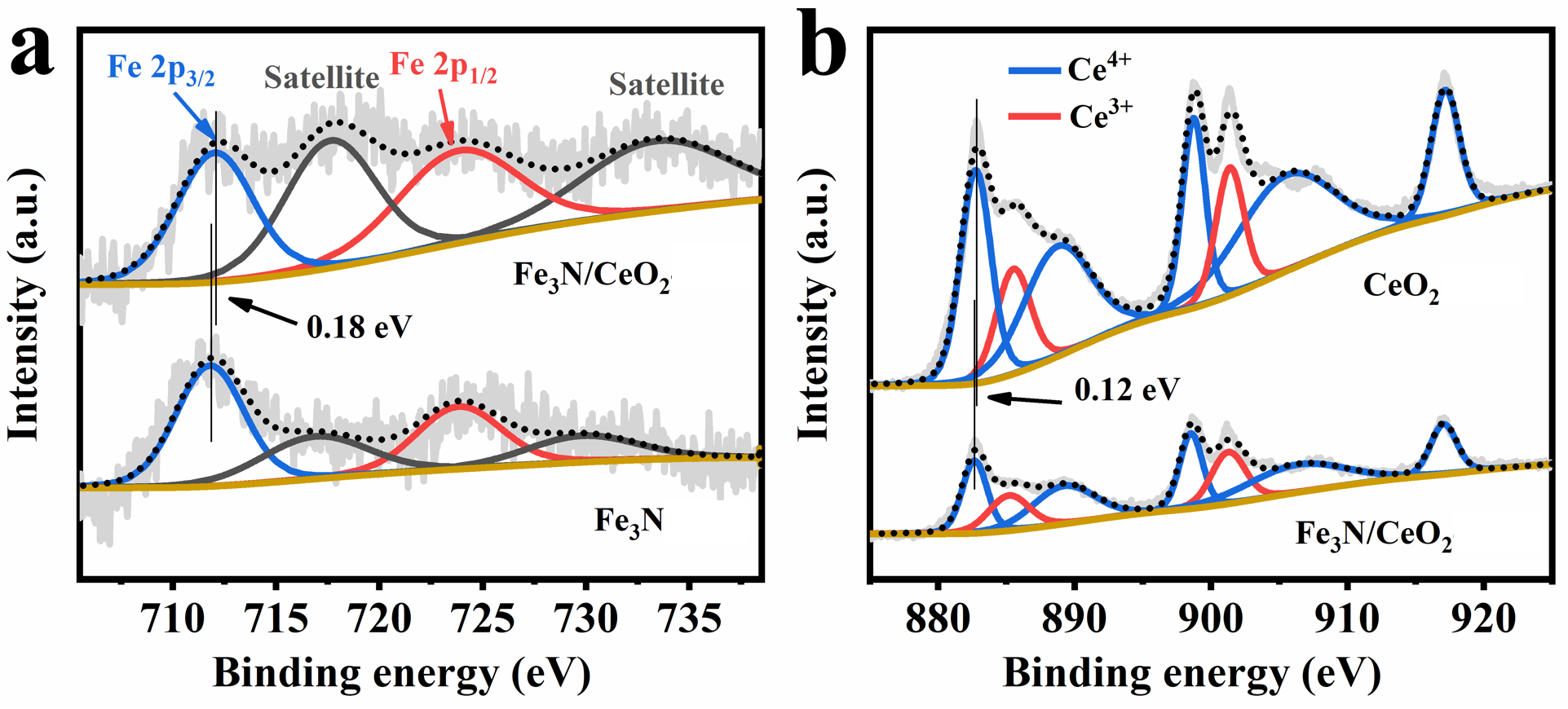


**Figure S5.** (a) XPS spectra of Fe 2p for Fe_3_N/CeO_2_ and Fe_3_N. (b) Ce 3d XPS spectra of Fe_3_N/CeO_2_ and CeO_2_.

By fitting the Fe 2p XPS spectra of Fe_3_N/CeO_2_ and Fe_3_N in Figure S3a, two characteristic peaks at 712.2 eV and 724.1 eV corresponding to Fe 2p_3/2_ and Fe 2p_1/2_, and two additional satellite peaks at 717.6 eV and 733.7 eV were observed.^[8]^ Meanwhile, the Ce 3d XPS spectra of Fe_3_N/CeO_2_ and CeO_2_ were also fitted as in Figure S3b. The seven component peaks can be divided into two groups, where the first group of peaks at 882.7, 889.4, 898.5, 906.9, and 917.2 eV corresponds to Ce^4+^, while the remaining two peaks at 885.3 and 901.5 eV correspond to Ce^3+^.^[9]^


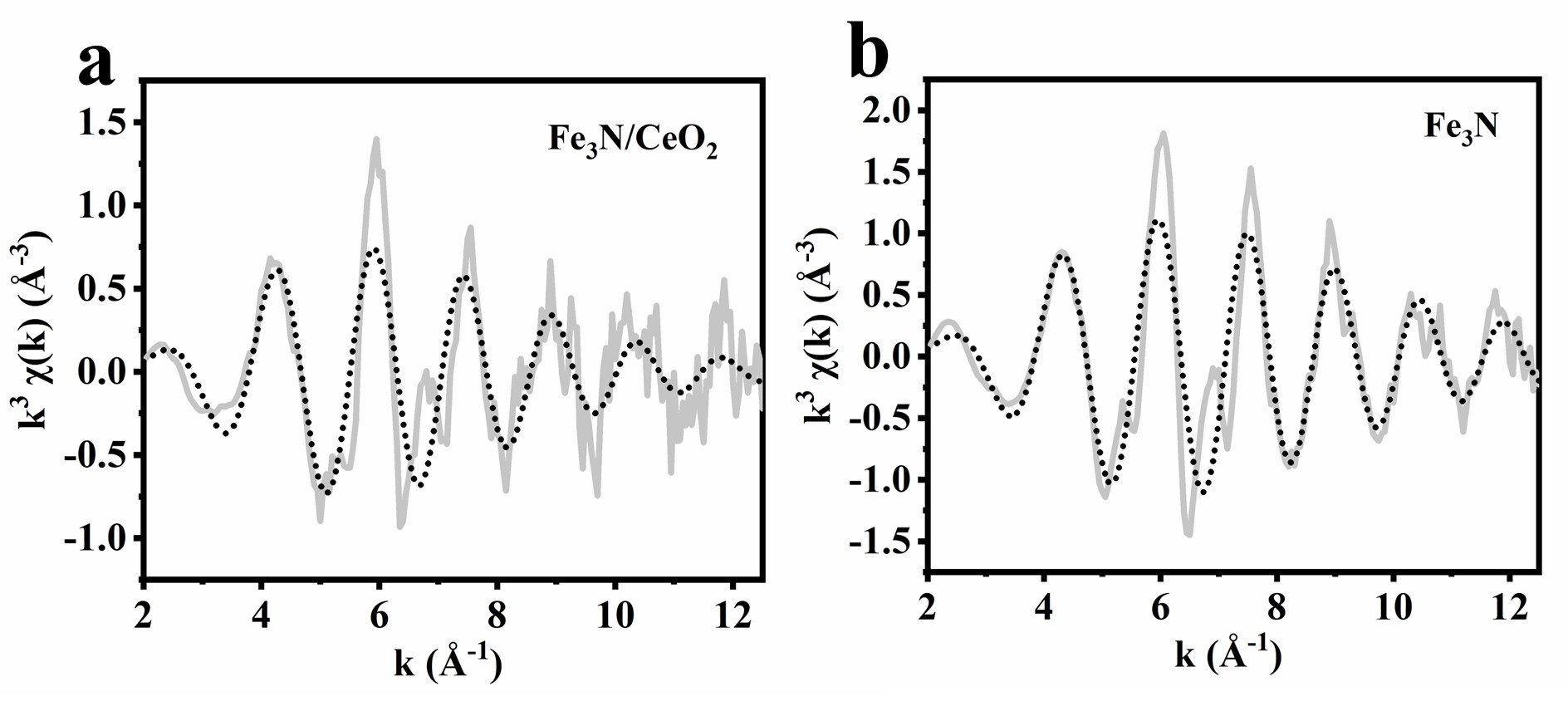


**Figure S6.** Fe K-edge EXAFS spectra of (a) Fe_3_N/CeO_2_ and (b) Fe_3_N at k space.


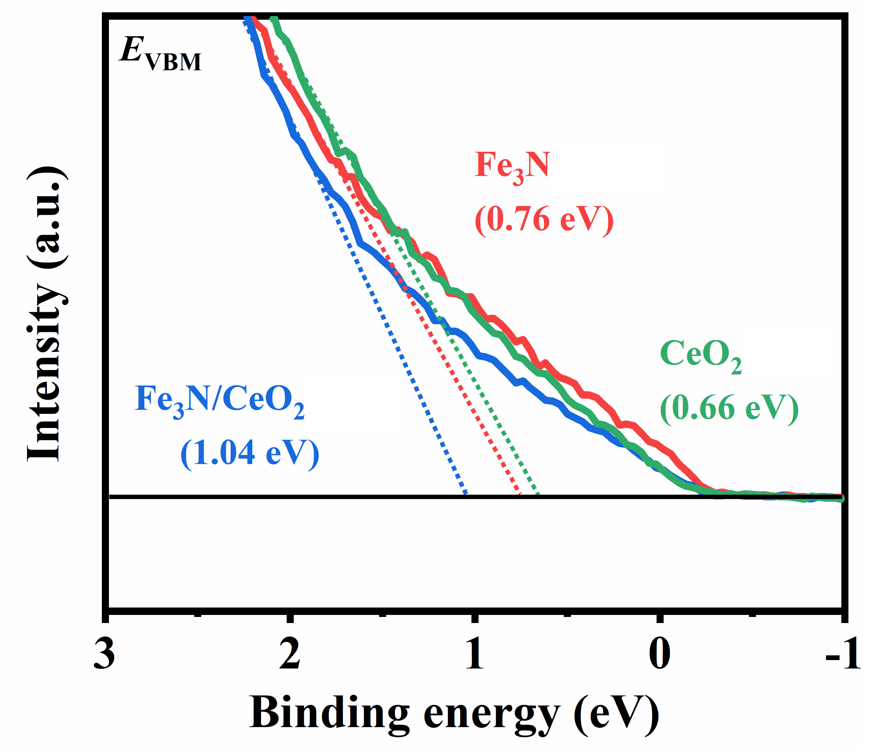


**Figure S7.** *E*_VBM_ of Fe_3_N/CeO_2_, Fe_3_N, and CeO_2_.

**

**

**Figure S8.** CV curves of Fe_3_N/CeO_2_, Fe_3_N, CeO_2_, and Fe_3_N@CeO_2_.


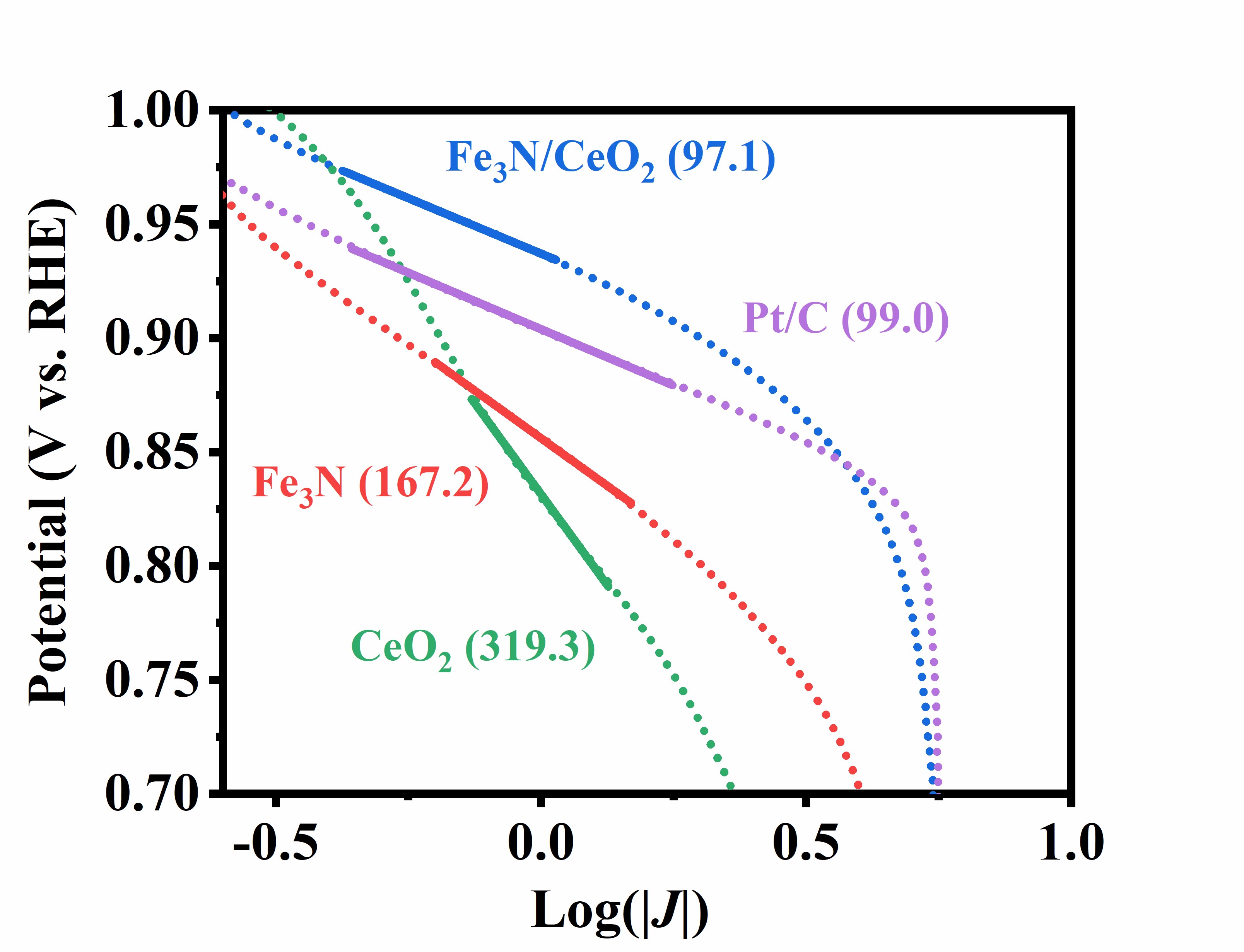


**Figure S9.** Fitted Tafel slopes of Fe_3_N/CeO_2_, Fe_3_N, CeO_2_, and Pt/C.


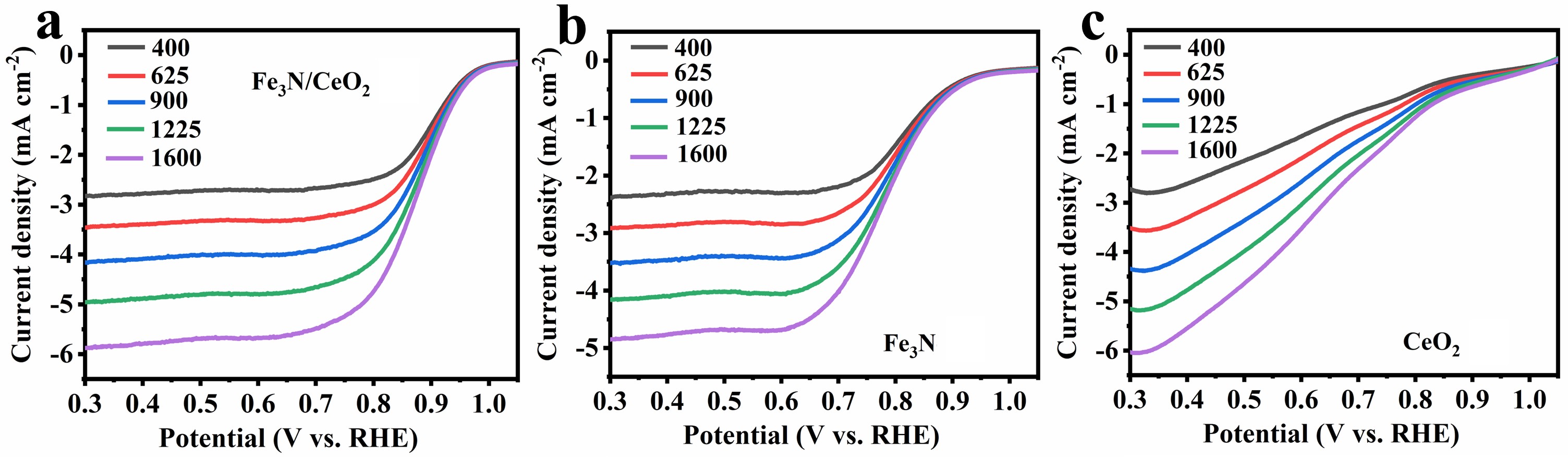


**Figure S10.** LSV curves with different rotating speeds of (a) Fe_3_N/CeO_2_, (b) Fe_3_N, and (c) CeO_2_.

By linearly fitting 1/|*J*| at 0.35 V (diffusion-controlled region) against *ω*^-1/2^ (*J* is the current density and *ω* is the RDE rotation speed), the *n* values (slopes) can be determined based on Koutecky-Levich (K-L) equation. Moreover, by attaining the intercepts of the K-L plots at *E*_onset_-0.05 V (polarization region), the ORR kinetic current densities can also be calculated.^[10]^


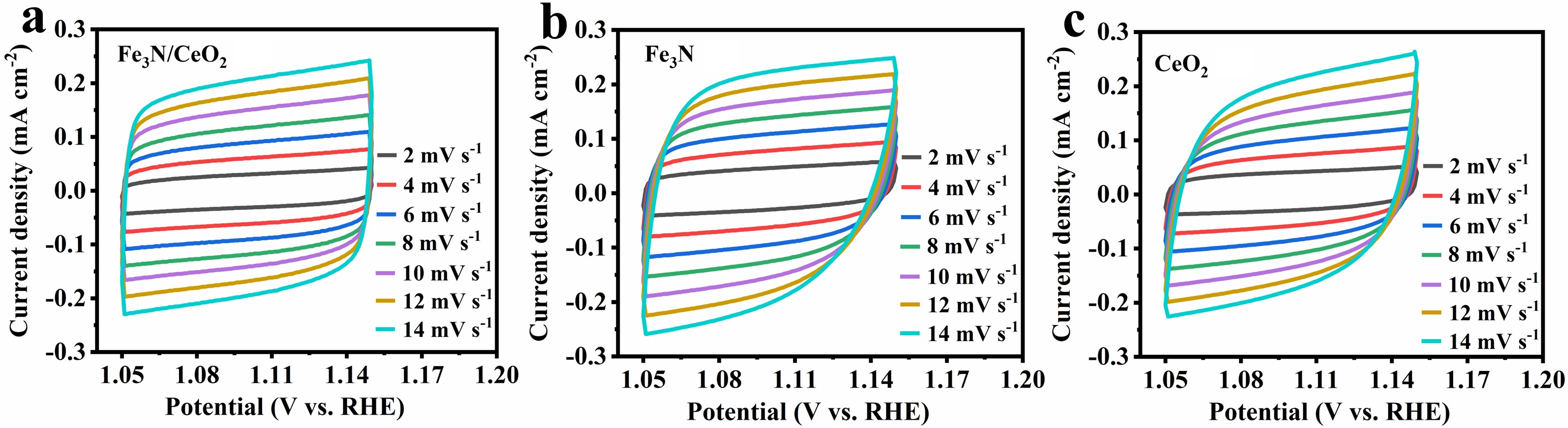


**Figure S11.** CV curves with different sweeping speeds of (a) Fe_3_N/CeO_2_, (b) Fe_3_N, and (c) CeO_2_ in the non-Faradaic region.

The electrochemically active surface areas (ECSA) of the materials were obtained by performing CV tests at different scan rates (2-14 mV s^-1^) in non-Faraday regions (Figure S8). By plotting the average of cathodic and anodic currents at 1.095 V against scan rate, the dielectric capacitances (*C*_dl_) of the materials via slopes can be obtained, after which the ECSAs can be calculated as follows:

ECSA = *C*_dl_ /*C*_s_

where *C*_s_ is the specific capacitance of an ideal flat surface (40 μF cm^-2^).

The ECSA-normalized kinetic current densities (*J*_k, ECSA_) were calculated as follows:

*J*_k, ECSA_= *J*_k_**A*/ECSA

where A is the geometric area of ​​the glassy carbon electrode (0.19625 cm^2^),

The turnover frequencies (TOFs) were calculated at 0.79 V as follows:

TOF=(*J***A*)/(4**F***M*)

where *J* is the current density at 0.79 V, *F* is the Faraday constant (96485 C mol^-1^), and *M* is the amount of metal sites on the electrode (Fe or Ce) in moles.


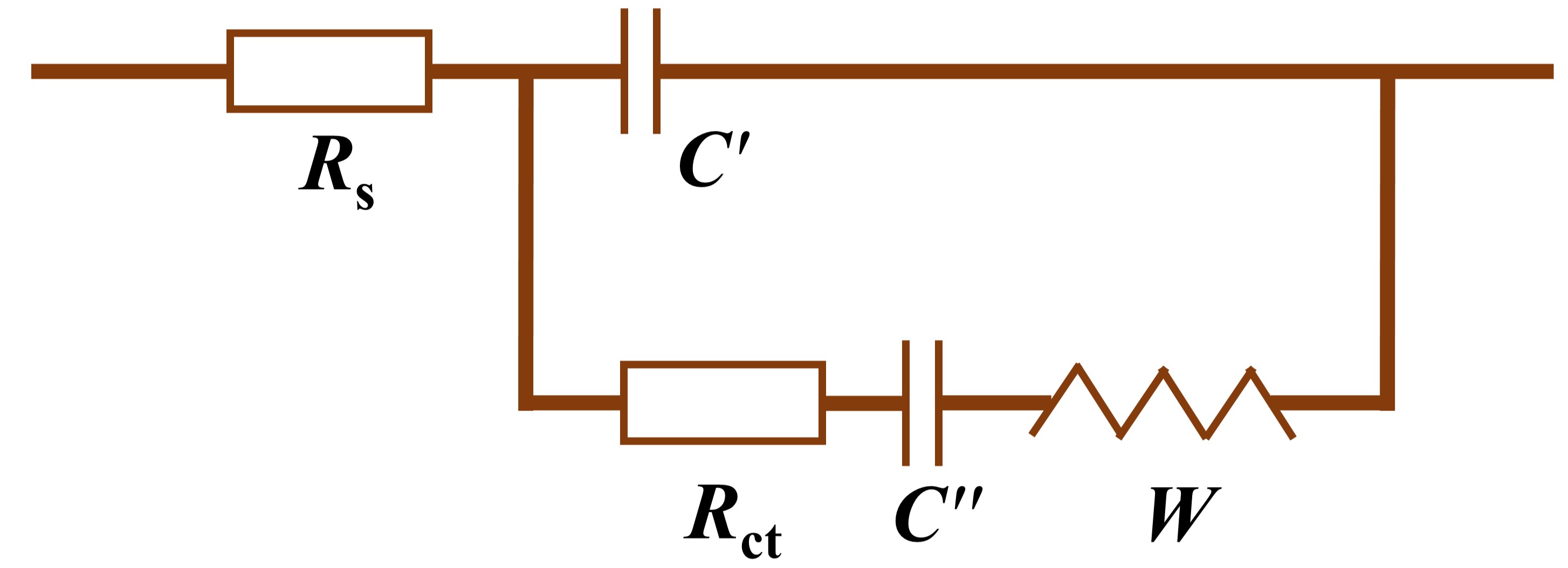


**Figure S12.** Equivalent circuit for EIS fitting.


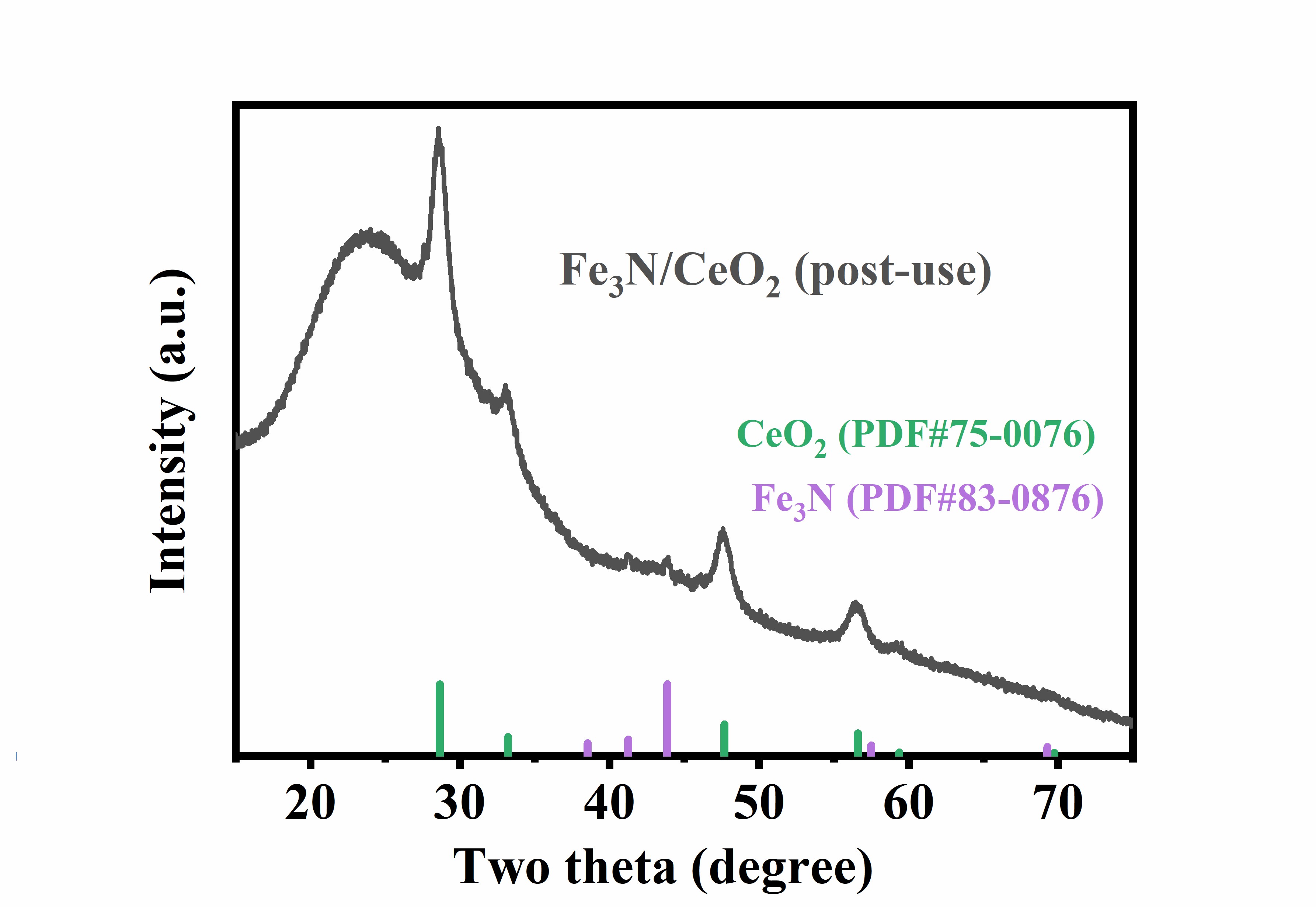


**Figure S13.** XRD pattern of post-use Fe_3_N/CeO_2_ after chronoamperometric test.


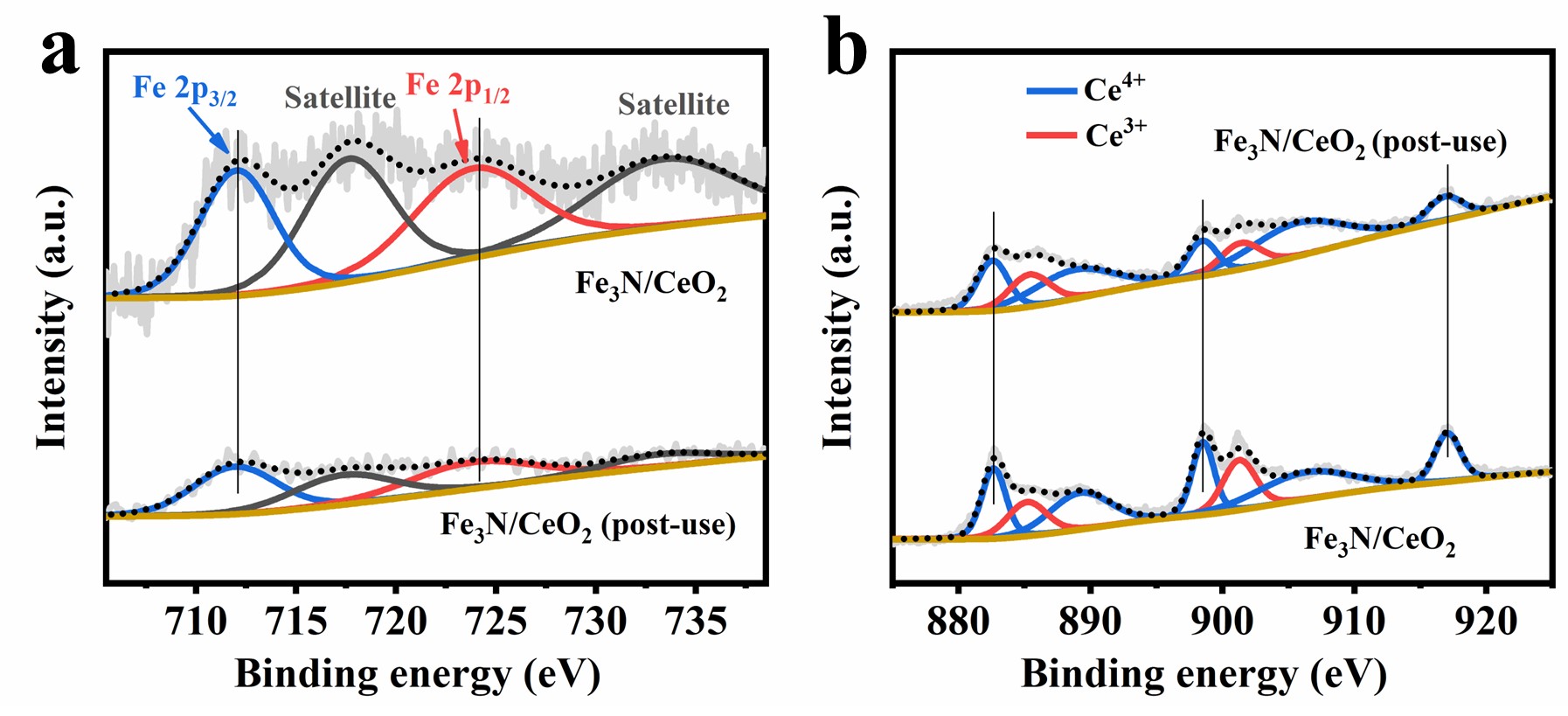


**Figure S14.** (a) Fe 2p and (b) Ce 3d XPS spectra of post-use Fe_3_N/CeO_2_ after chronoamperometric test (counterpart: fresh Fe_3_N/CeO_2_).


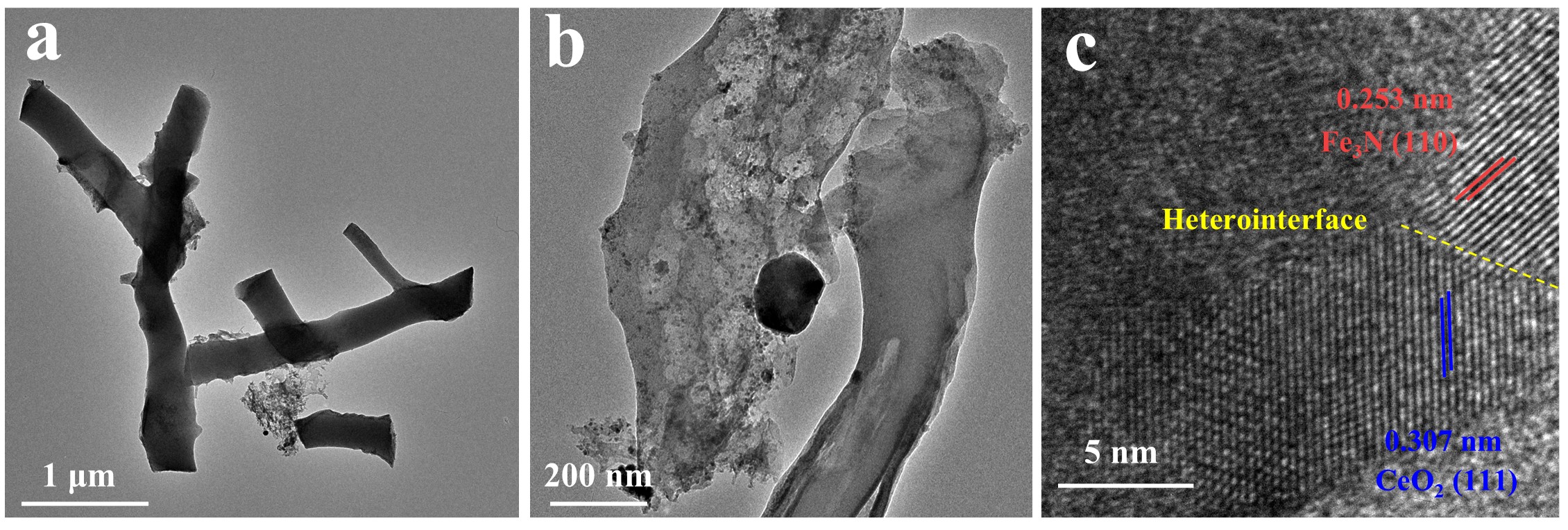
**Figure S15.** (a, b) TEM and (c) HRTEM images of post-use Fe_3_N/CeO_2_ after chronoamperometric test.


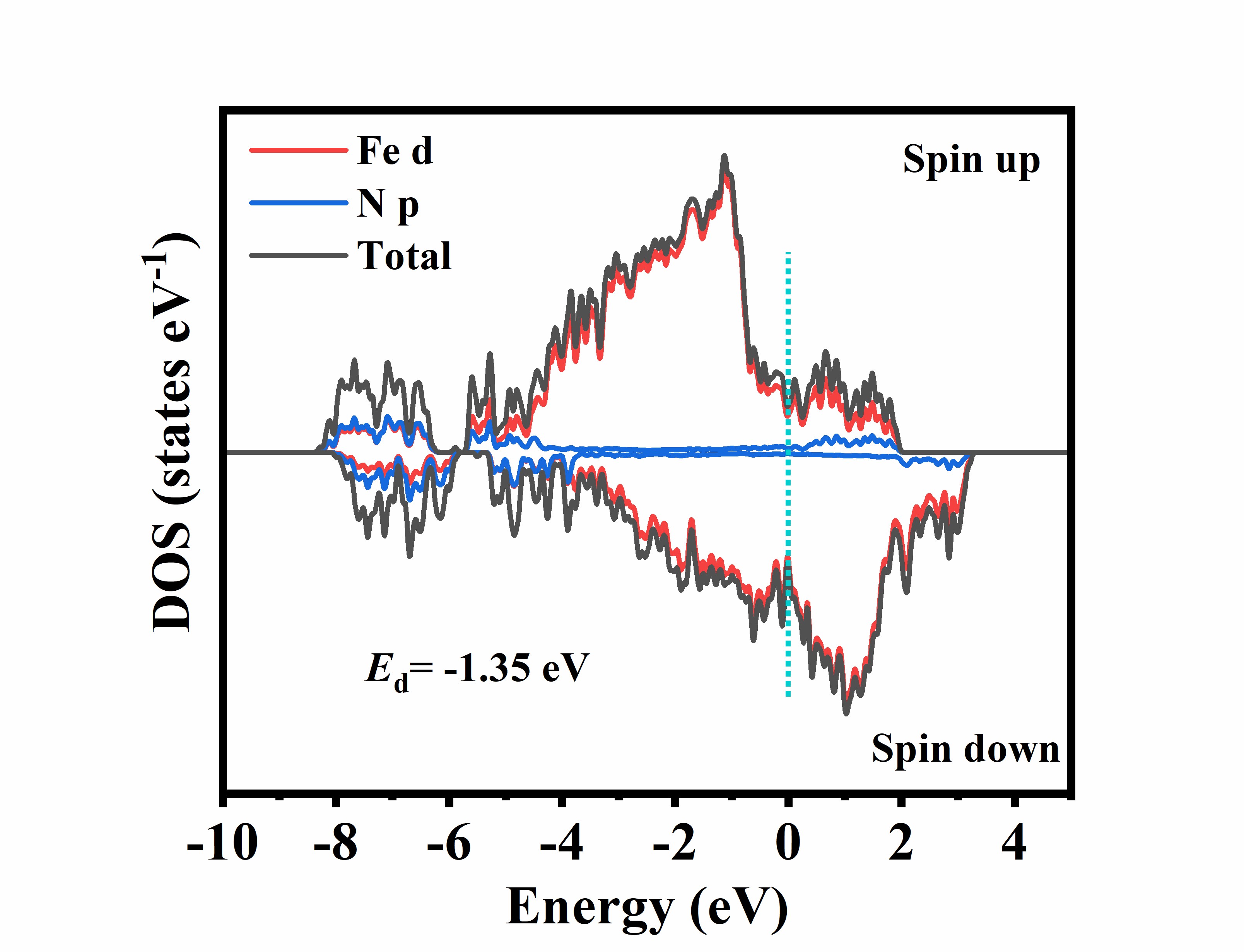


**Figure S16.** Density of states of Fe_3_N.


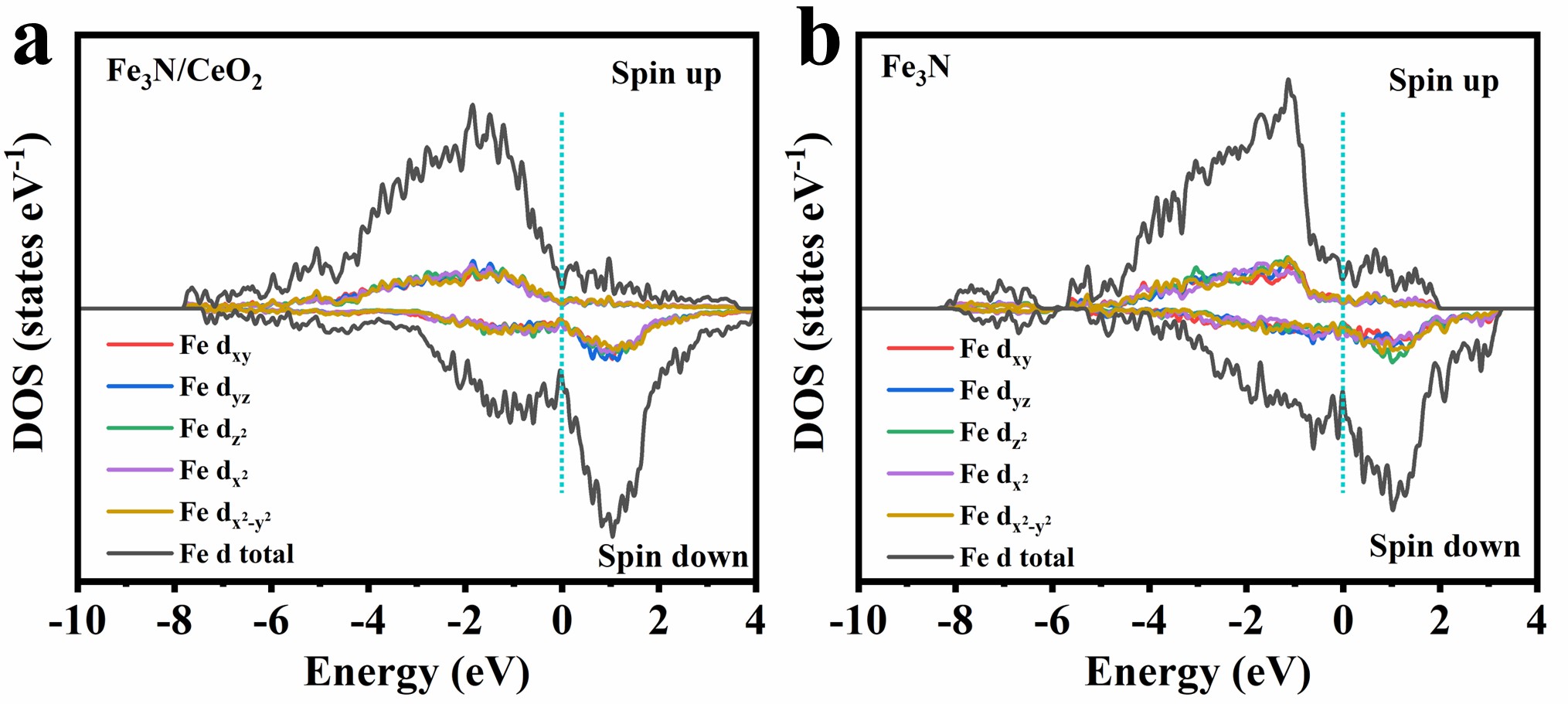


**Figure S17.** Degenerate Fe 3d states of (a) Fe_3_N/CeO_2_ and (b) Fe_3_N.

The total number of d-electrons can be determined by integrating over the entire d-orbital. The number of occupied d-electrons is calculated by integrating up to the Fermi level. Unpaired electrons can be obtained by subtracting the occupied d-electrons from the total d-electrons. Furthermore, the difference between spin-up and spin-down d-electrons can be calculated by taking the absolute value of spin-up d-electrons minus spin-down occupied d-electrons.


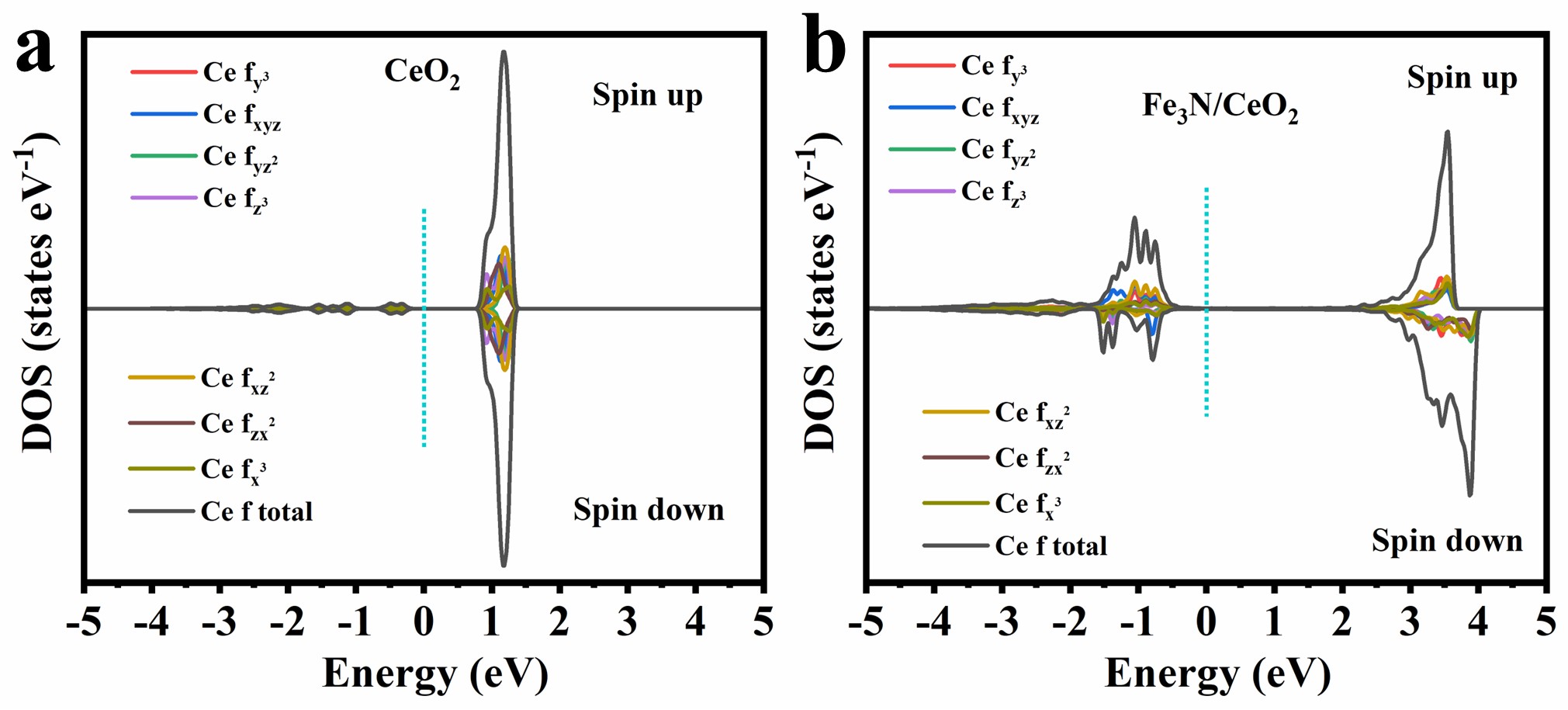


**Figure S18.** Degenerate Ce 4f states of (a) CeO_2_ and (b) Fe_3_N/CeO_2_.


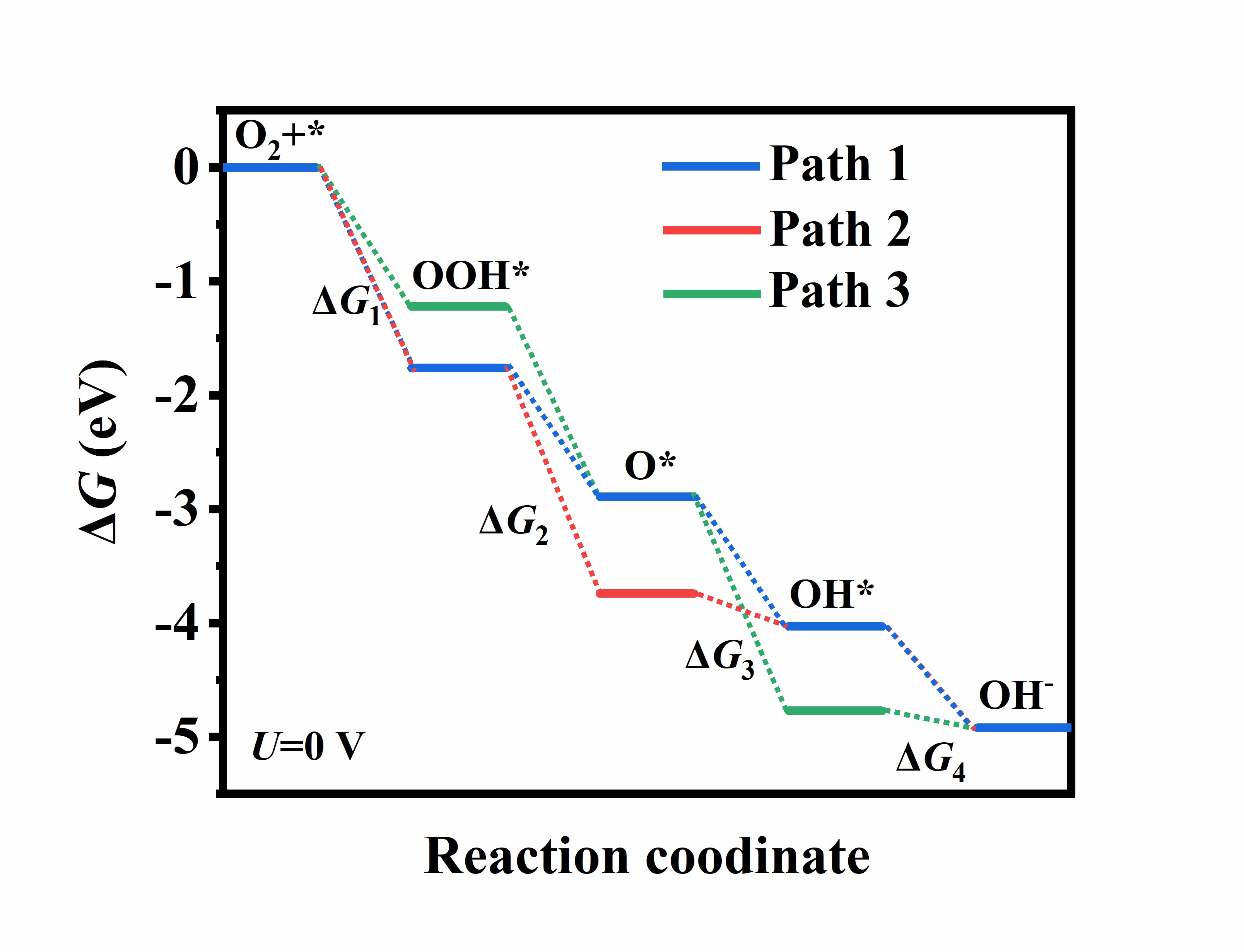


**Figure S19.** Free energy diagram at *U*=1.23 V of different ORR paths.

**Table S1.** Fitting results of Fe K-edge Fourier transform EXAFS curves of Fe_3_N/CeO_2_ and Fe_3_N.

| **Sample** | **Edge** | **Path** | ***N*** | ***R*(Å)** | ***σ*^2^(Å^2^)** | **Δ*E*_0_** | **R-factor** |
| --- | --- | --- | --- | --- | --- | --- | --- |
| Fe_3_N/CeO_2_ | Fe | Fe-N(O) | 5.75 | 2.05 | 0.00953 | 2.7 | 0.0192 |
| Fe_3_N | Fe | Fe-N | 5.99 | 2.10 | 0.00612 | 2.6 | 0.0098 |

**Table S2.** *E*_onset_ and *E*_1/2_ values of various recently reported heterostructural electrocatalysts for ORR.

| Sample | *E*_onset_ | *E*_1/2_ | References |
| --- | --- | --- | --- |
| c-CoSe_2_-CoN/NC | 0.97 | 0.85 | ^[11]^ |
| NiFe_2_O_4_/TiO_2_ | 0.97 | 0.84 | ^[12]^ |
| Fe_20_Ni_21_Co_23_\|Mn_11_Ga_24_O_x_/CNT | 0.921 | 0.824 | ^[13]^ |
| J-CeO_2_/ZCS | 0.95 | 0.87 | ^[14]^ |
| P-NCO/NCN-CF@CC | 0.973 | 0.828 | ^[15]^ |
| CCSO/NC-2 | 0.88 | 0.75 | ^[16]^ |
| Fe_2_NiO_4_/FeNiS_2_ MTs | 0.97 | 0.87 | ^[17]^ |
| Co/Co_2_P@PNCF | 0.94 | 0.87 | ^[18]^ |
| Gd_2_O_3_-Co/NG | 0.93 | 0.82 | ^[19]^ |
| Fe_3_N/CeO_2_ | 0.986 | 0.874 | This work |

**Table S3.** Output data of EIS fitting.

| Sample | *R*_s_ (ohm) | *R*_ct_ (ohm) | *C'*  (F) | *C''*  (F) | *W* (S*s^1/2^) |
| --- | --- | --- | --- | --- | --- |
| Fe_3_N/CeO_2_ | 38.6 | 2.1 | 7.26*10^-5^ | 3.70*10^-3^ | 3.76*10^-2^ |
| Fe_3_N | 39.3 | 7.9 | 3.18*10^-4^ | 1.52*10^-3^ | 1.01*10^-2^ |
| CeO_2_ | 38.9 | 4.7 | 1.07*10^-5^ | 1.22*10^-2^ | 2.27*10^-2^ |

**Table S4.** Maximum power densities of various recently reported metal-air batteries with heterostructural electrocatalysts.

| Sample | Anode \| electrolyte | Maximum power density (mW cm^-2^) | References |
| --- | --- | --- | --- |
| Fe_20_Ni_21_Co_23_\|Mn_11_Ga_24_O_x_/CNT | Zn foil \| 6.0 M KOH and 0.2 M Zn(CH_3_COO)_2_ | 136.1 mW cm**^-^**^2^ | ^[13]^ |
| Co/CoSe@NC | Zn foil \| 6.0 M KOH and 0.2 M Zn(CH_3_COO)_2_ | 145 mW cm**^-^**^2^ | ^[20]^ |
| J−CeO_2_/ZCS | Zn foil \| 6.0 M KOH and 0.2 M Zn(CH_3_COO)_2_ | 168.7 mW cm**^-^**^2^ | ^[14]^ |
| Eu_2_O_3_-Co/NC | Zn plate \| 6 M KOH | 123.3 mW cm**^-^**^2^ | ^[21]^ |
| Fe_2_NiO_4_/FeNiS_2_ MTs | Zn foil \| 6.0 M KOH and 0.2 M Zn(CH_3_COO)_2_ | 81.74 mW cm**^-^**^2^ | ^[17]^ |
| CMNC-1 | Al-Mg-Sn \| 4 M NaOH | 139.8 mW cm**^-^**^2^ | ^[22]^ |
| CoP@N,P-CNFs | Al foil \| 6 M KOH | 72.8 mW cm**^-^**^2^ | ^[23]^ |
| Ag/Mn_3_O_4_ | Al-Mg-Sn \| 4 M NaOH | 148.3 mW cm**^-^**^2^ | ^[7]^ |
| Ag@MnO_2_/MWNTs | Al foil \| 4 M KOH | 140.2 mW cm**^-^**^2^ | ^[24]^ |
| Fe_3_N/CeO_2_ | Al-Mg-Sn \| 4 M NaOH | 157.8 mW cm**^-^**^2^ | This work |

**References**

[1] a) G. Kresse, J. Furthmüller, *Comput. Mater. Sci.* **1996**, 6, 15; b) G. Kresse, J. Furthmüller, *Phys. Rev. B* **1996**, 54, 11169; c)J. P. Perdew, K. Burke, M. Ernzerhof, *Phys. Rev. Lett.* **1996**, 77, 3865.

[2] a) G. Kresse, D. Joubert, *Physical Review B* **1999**, 59, 1758; b) P. E. Blöchl, *Phys. Rev. B* **1994**, 50, 17953.

[3] H. J. Monkhorst, J. D. Pack, *Phys. Rev. B* **1976**, 13, 5188.

[4] a) S. Grimme, J. Antony, S. Ehrlich, H. Krieg, *J. Chem. Phys.* **2010**, 132, 154104; b) S. Grimme, S. Ehrlich, L. Goerigk, *J. Comput. Chem.* **2011**, 32, 1456.

[5] V. Wang, N. Xu, J.-C. Liu, G. Tang, W.-T. Geng, *Comput. Phys. Commun.* **2021**, 267, 108033.

[6] a) E. Sanville, S. D. Kenny, R. Smith, G. Henkelman, *J. Comput. Chem.* **2007**, 28, 899; b) W. Tang, E. Sanville, G. Henkelman, *J Phys Condens Matter* **2009**, 21, 084204; c) G. Henkelman, A. Arnaldsson, H. Jónsson, *Comput. Mater. Sci.* **2006**, 36, 354; d) M. Yu, D. R. Trinkle, *J. Chemical Phys.* **2011**, 134, 064111.

[7] R. Cheng, K. Li, H. Li, F. Sun, X. He, T. Zhao, J. Zhang, C. Fu, *Nano Res.* **2024**, 17, 3622.

[8] B. Zhang, J. Zhao, H. Qiu, M. Chen, X. Ren, H. Wang, Q. Wei, *ChemPhysChem* **2024**, 25, e202400738.

[9] M. Li, X. Wang, K. Liu, Z. Zhu, H. Guo, M. Li, H. Du, D. Sun, H. Li, K. Huang, Y. Tang, G. Fu, *Adv. Energy Mater.* **2023**, 13, 2301162.

[10] R. Cheng, K. Li, Z. Li, M. Jiang, F. Wang, Z. Yang, T. Zhao, P. Meng, C. Fu, *J. Power Sources* **2023**, 556, 232476.

[11] X. Xu, X. Wang, S. Huo, X. Liu, X. Ma, M. Liu, J. Zou, *Adv. Mater.* **2024**, 36, 2306844.

[12] S. Palanisamy, M. Gopalakrishnan, S. Ingavale, M. Etesami, W. Limphirat, W.-R. Liu, M. Tipplook, K. Teshima, S. Kheawhom, *J. Energy Storage* **2024**, 98, 113139.

[13] L. Luo, Y. Liu, S. Chen, Q. Zhu, D. Zhang, Y. Fu, J. Li, J. Han, S. Gong, *Small* **2024**, 20, 2308756.

[14] J. Zhang, X. Dong, G. Wang, J. Chen, R. Wang, *Appl. Catal. B Environ.* **2024**, 342, 123459.

[15] Y. Liu, Z. Jiang, Z.-J. Jiang, *Adv. Funct. Mater.* **2023**, 33, 2302883.

[16] J. Cai, H. Zhang, L. Zhang, Y. Xiong, T. Ouyang, Z.-Q. Liu, *Adv. Mater.* **2023**, 35, 2303488.

[17] X. Li, D. C. Nguyen, K. Dong, S. Prabhakaran, D. T. Tran, D. H. Kim, N. H. Kim, J. H. Le, *Chem. Eng. J.* **2024**, 489, 151210.

[18] Z. Xu, J. Chen, T. Zhang, H. Lu, L. Yan, J. Ning, Y. Hu, *Adv. Energy Mater.* **2025**, 15, 2402839.

[19] M. Li, Y. Wang, Y. Zheng, G. Fu, D. Sun, Y. Li, Y. Tang, T. Ma, *Adv. Energy Mater.* **2020**, 10, 1903833.

[20] K. Li, R. Cheng, Q. Xue, P. Meng, T. Zhao, M. Jiang, M. Guo, H. Li, C. Fu, *Chem. Eng. J.* **2022**, 450, 137991.

[21] X. Wang, J. Wang, P. Wang, L. Li, X. Zhang, D. Sun, Y. Li, Y. Tang, Y. Wang, G. Fu, *Adv. Mater.* **2022**, 34, 2206540.

[22] K. Li, R. Cheng, Q. Xue, T. Zhao, F. Wang, C. Fu, *ACS Appl. Mater. Interfaces* **2023**, 15, 9150.

[23] J. Liu, C. Zhang, S. Yuan, W. Yang, Y. Cao, J. Deng, B. Xu, H. Lu, *Chem. Eng. J.* **2022**, 428, 131326.

[24] Y. Zhang, L. Yin, Z. Luo, X. Zhuge, P. Wei, Z. Song, K. Luo, *Sustain. Energy Fuels* **2023**, 7, 3276.
